# Supplementary material for: Evolution-inspired engineering of anthracycline methyltransferases
Source: PNAS Nexus. 2023 Feb 28;2(2):pgad009. doi: 10.1093/pnasnexus/pgad009 (PMC9976750; doi:10.1093/pnasnexus/pgad009)
Supplement: pgad009_Supplementary_Data [file pgad009_supplementary_data.docx]

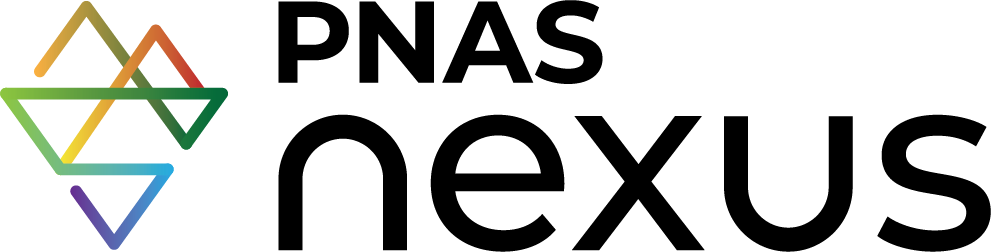


**Supplementary Information for**

Evolution-Inspired Engineering of Anthracycline Methyltransferases.

Authors

Pedro Dinis,^1^ Heli Tirkkonen,^1^ Benjamin Nji Wandi, Vilja Siitonen, Jarmo Niemi, Thadée Grocholski and Mikko Metsä-Ketelä,*

Affiliations

Department of Life Technologies, University of Turku, FIN-20014 Turku, Finland

^1^ equal contribution

Contact*

E-mail: mianme@utu.fi, Tel: +35823336846, Fax: +35822317666

Keywords

Enzyme evolution; protein engineering; Streptomyces; polyketide; natural product

**Supplementary Figures**


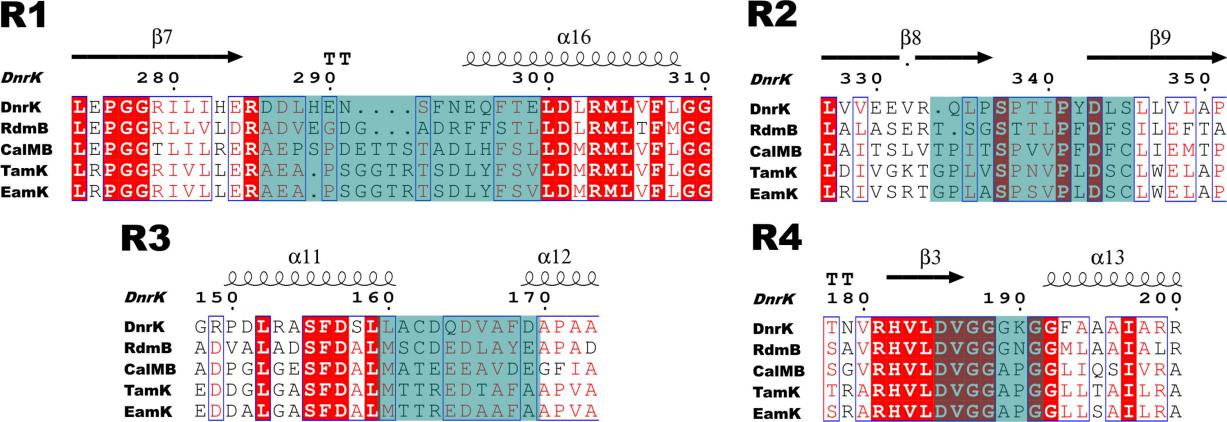


**Fig. S1. Multiple sequence alignment (ClustalOmega**(^[[1]](#endnote-1)^)**, ENDScript**(^[[2]](#endnote-2)^)**) of the relevant members of the family, with chimeric regions highlighted (teal).**


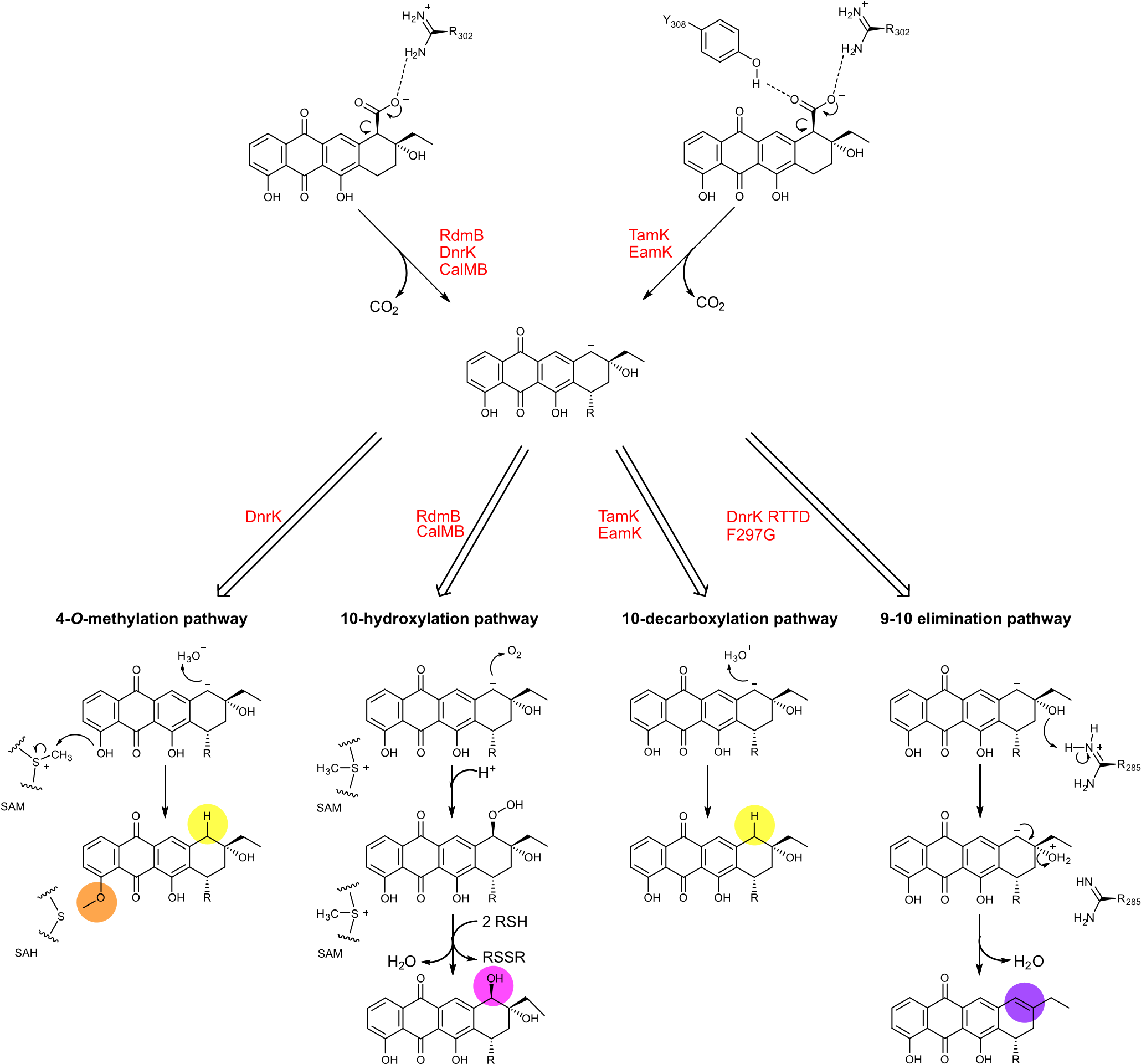


**Fig. S2. Reaction mechanisms of all the enzymatic reactions presented in this study.** All the reaction pathways share a 10-decarboxylation step leading to a common carbanion intermediate, which is repeated in the different diverging reaction pathways for clarity. The enzyme names are marked in red along the pathways.


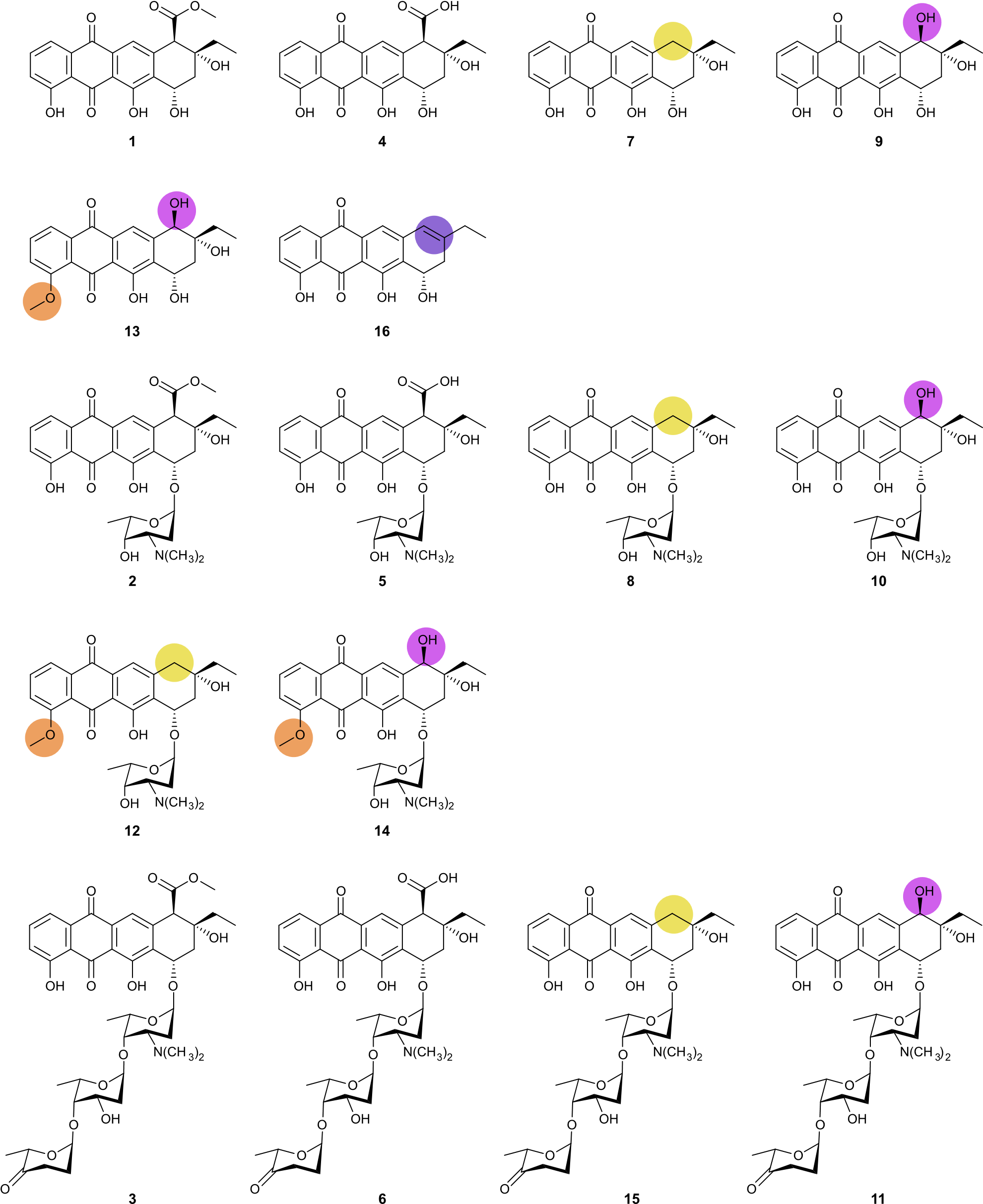


**Fig. S3.** **Chemical structures present in this study.**


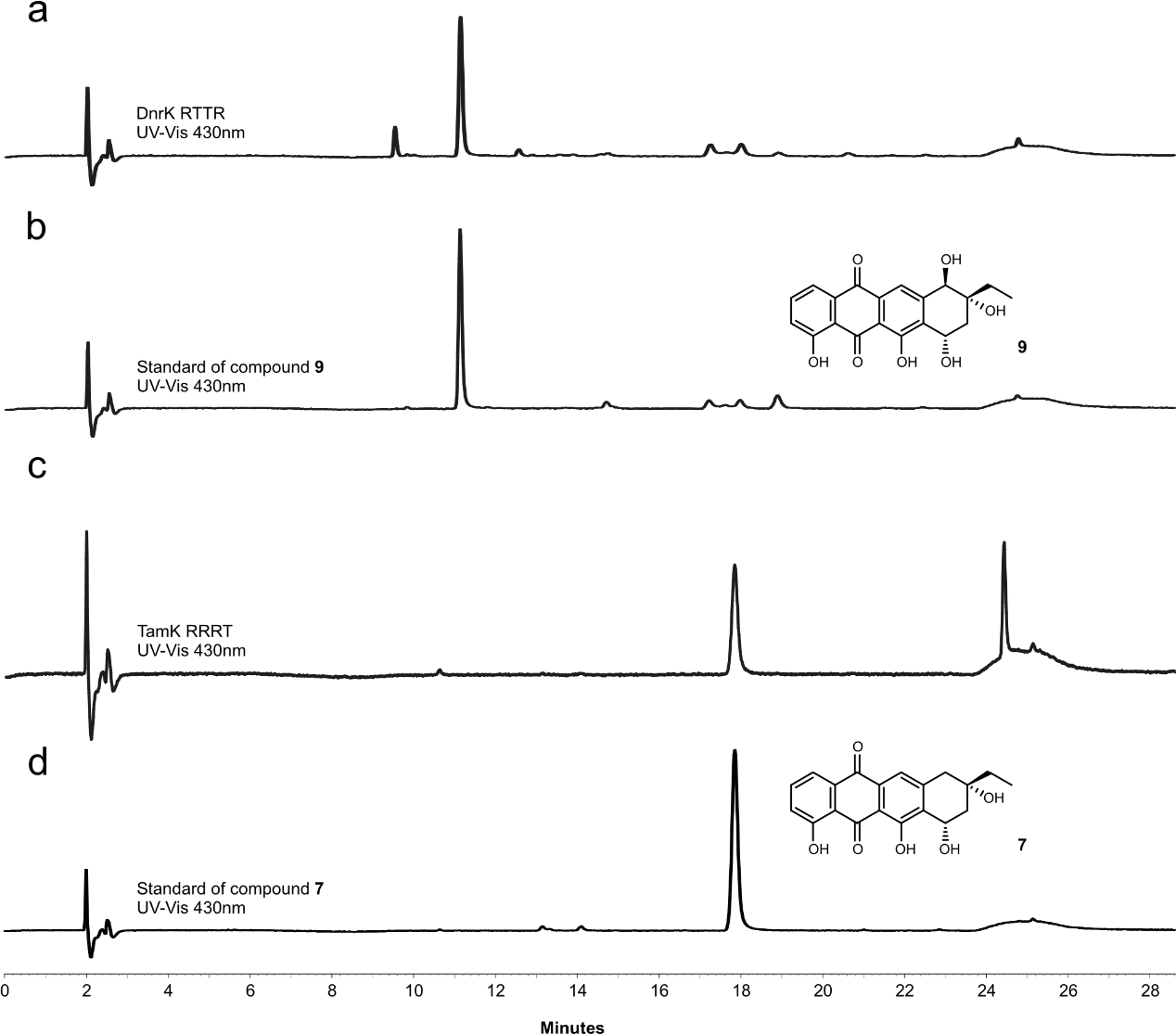


**Fig. S4.** **HPLC comparisons of enzymatic reactions products from 4 as a substrate with standard compounds.** UV-Vis chromatogram traces were recorded at 430 nm. **a,** Enzymatic reaction products by DnrK RTTR. **b,** Standard of compound **9** obtained by enzymatic reaction of RdmB with **4** as a substrate. **c,** Enzymatic reaction products by TamK RRRT. **d,** Standard of compound **7** obtained by enzymatic reaction of TamK with **4** as a substrate.


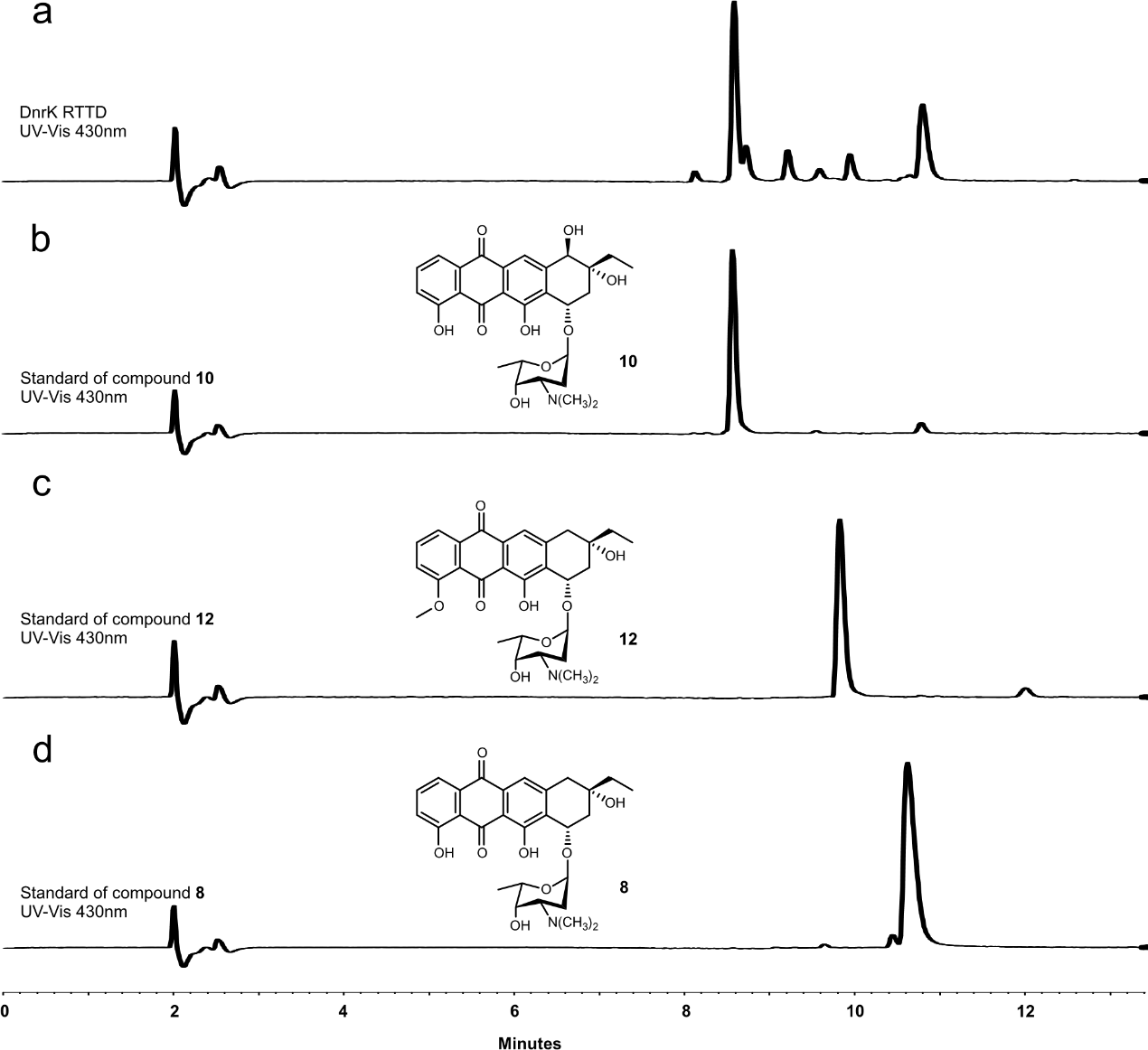


**Fig. S5.** **HPLC comparisons of enzymatic reactions products from 5 as a substrate with standard compounds.** UV-Vis chromatogram traces were recorded at 430 nm. **a,** Enzymatic reaction products by DnrK RTTD. **b,** Standard of compound **10** obtained by enzymatic reaction of RdmB with **5** as a substrate. **c,** Standard of compound **12** obtained by enzymatic reaction of DnrK with **5** as a substrate. **d,** Standard of compound **8** obtained by enzymatic reaction of TamK with **5** as a substrate.


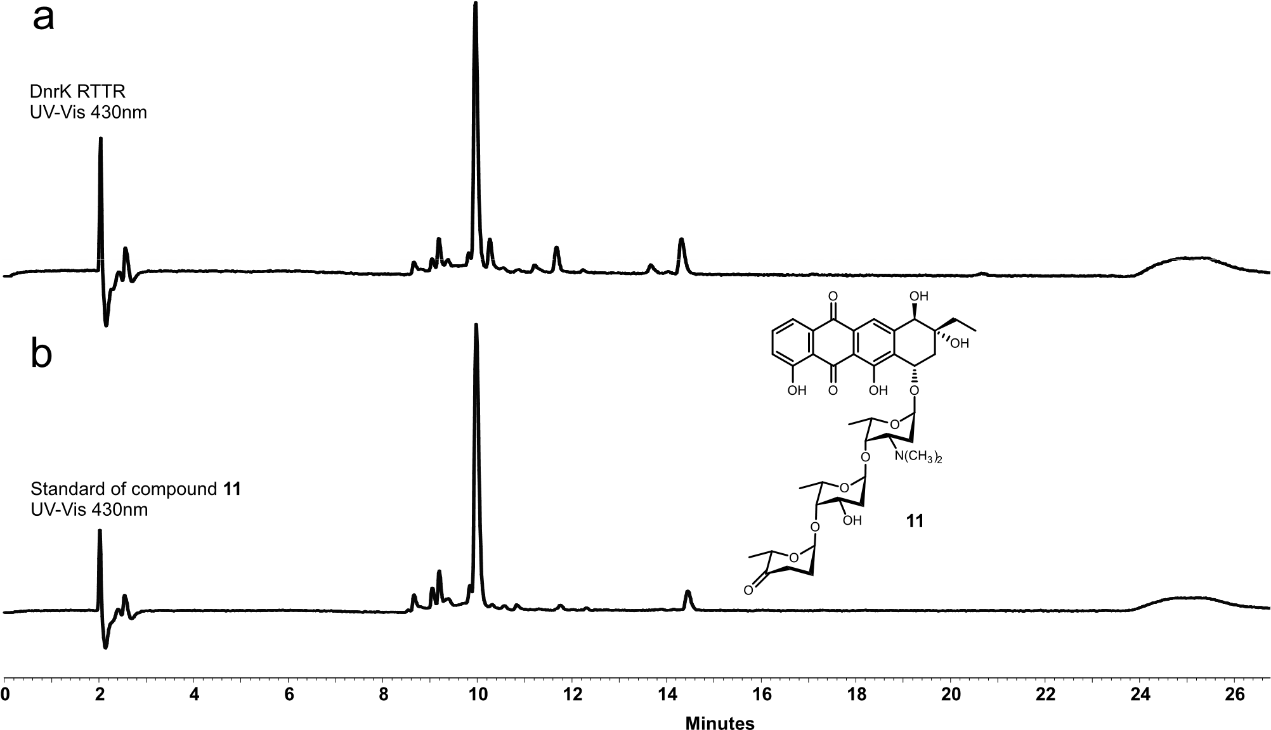


**Fig. S6.** **HPLC comparisons of enzymatic reactions products from 6 as a substrate with standard compounds**. UV-Vis chromatogram traces were recorded at 430 nm. **a,** Enzymatic reaction products by DnrK RTTR. **b,** Standard of compound **11** obtained by enzymatic reaction of RdmB with **6** as a substrate.


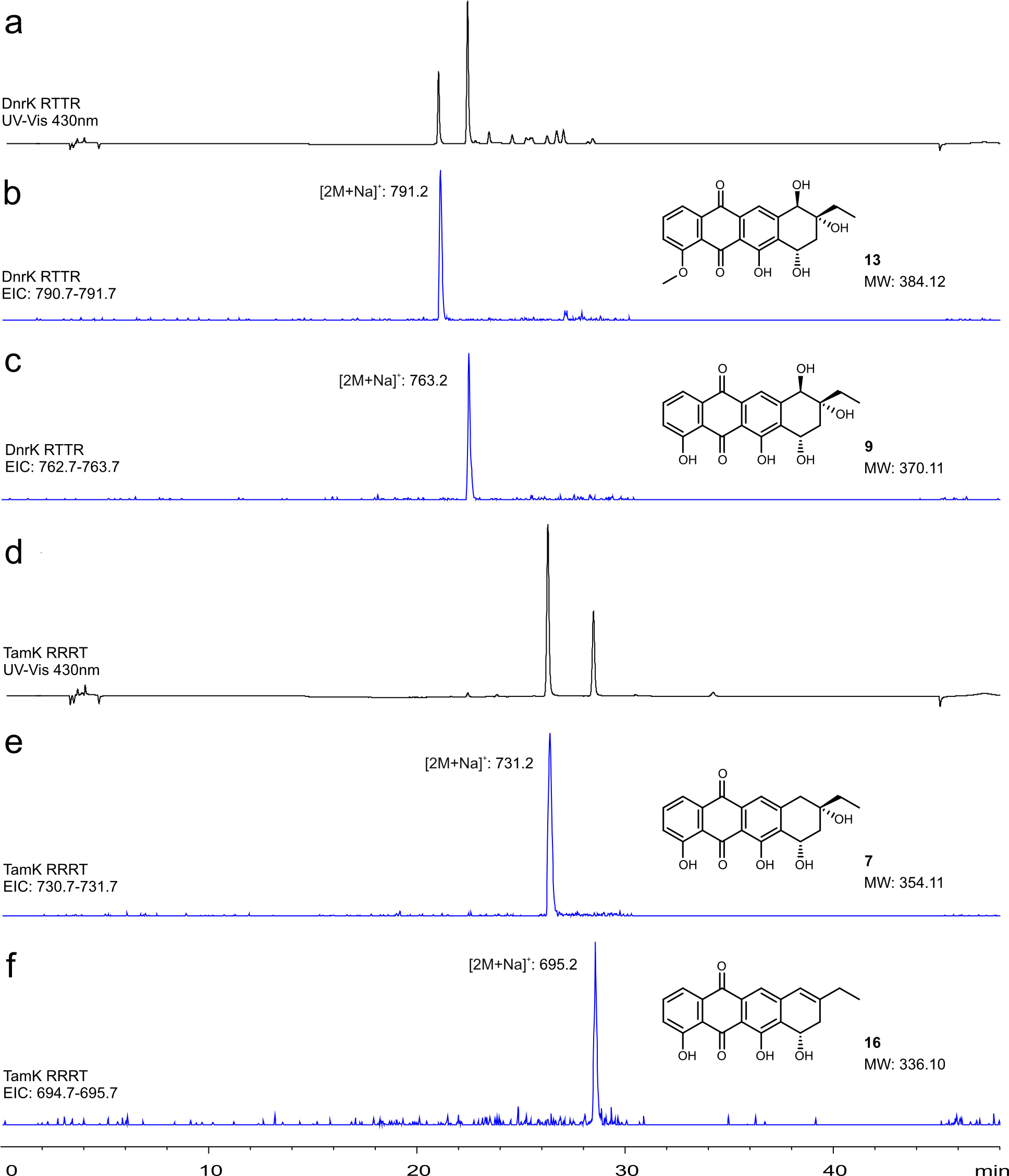


**Fig. S7. LC-MS analysis of enzymatic reactions with 4 as a substrate. a,** UV-Vis chromatogram trace recorded at 430 nm for enzymatic reaction products of DnrK RTTR. **b** **and** **c,** Extracted ion chromatogram traces in positive mode for DnrK RTTR products. **d,** UV-Vis chromatogram trace recorded at 430 nm for enzymatic reaction products of TamK RRRT. **e** **and** **f,** Extracted ion chromatogram traces in positive mode for TamK RRRT products. Products are observed as sodium adducts [2M+Na]^+^ under the conditions used, giving consistently values of 2M + 22.99.


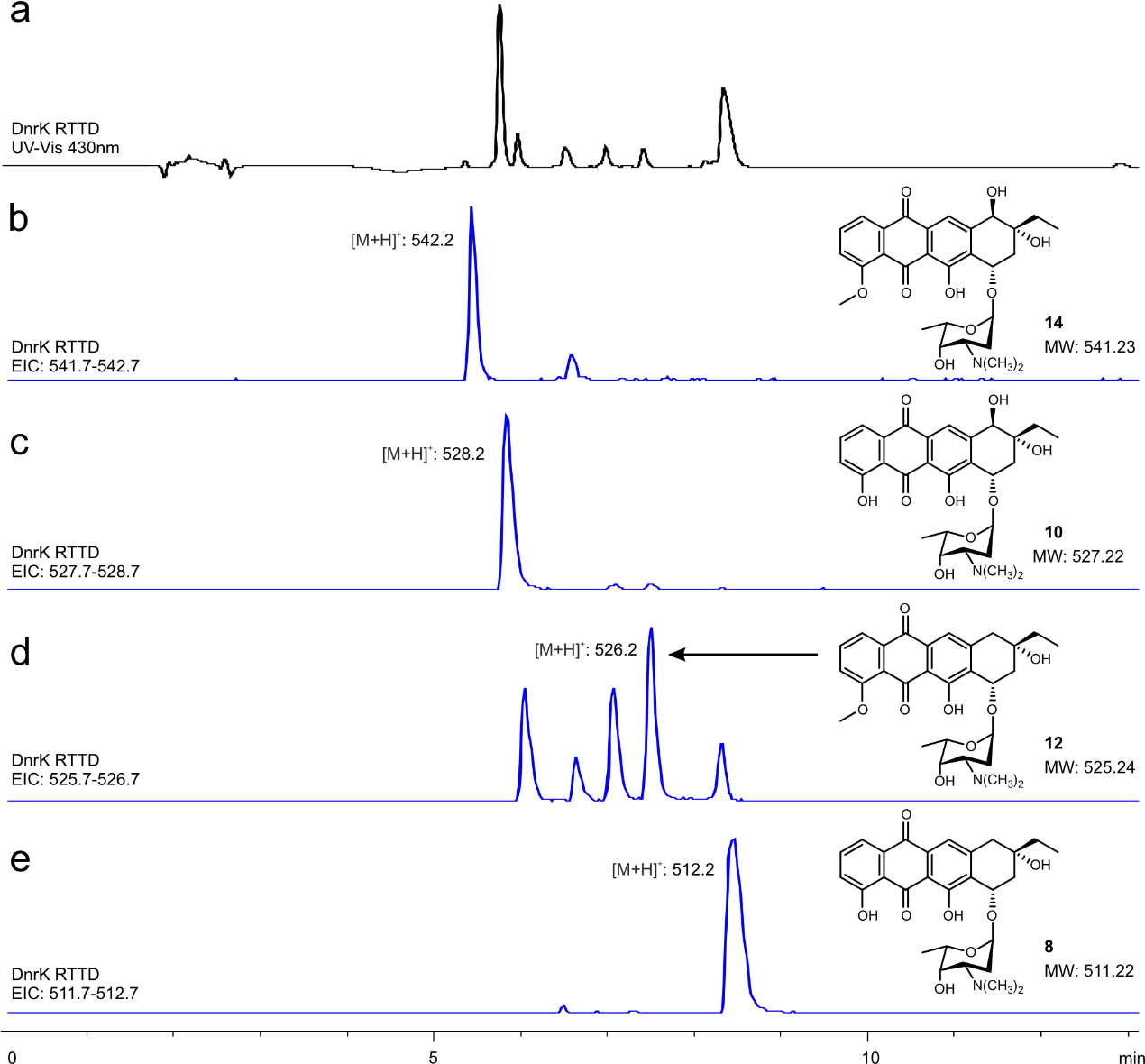


**Fig. S8. LC-MS analysis of enzymatic reactions with 5 as a substrate. a,** UV-Vis chromatogram trace recorded at 430 nm for enzymatic reaction products of DnrK RTTD. **b**, **c, d, and e,** Extracted ion chromatogram traces in positive mode for DnrK RTTD products.


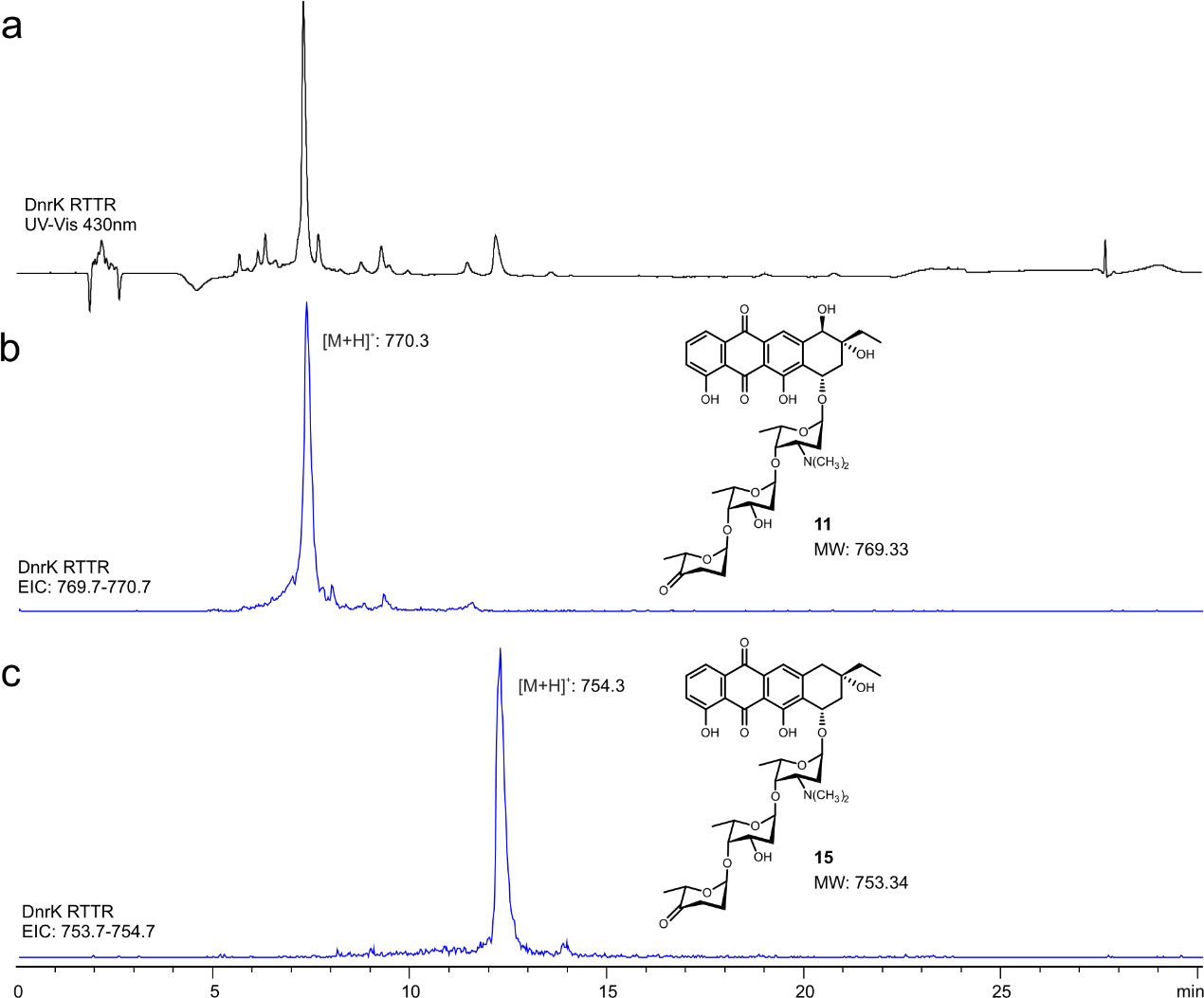


**Fig. S9. LC-MS analysis of enzymatic reactions with 6 as a substrate. a,** UV-Vis chromatogram trace recorded at 430 nm for enzymatic reaction products of DnrK RTTR. **b** **and** **c,** Extracted ion chromatogram traces in positive mode for DnrK RTTR products.


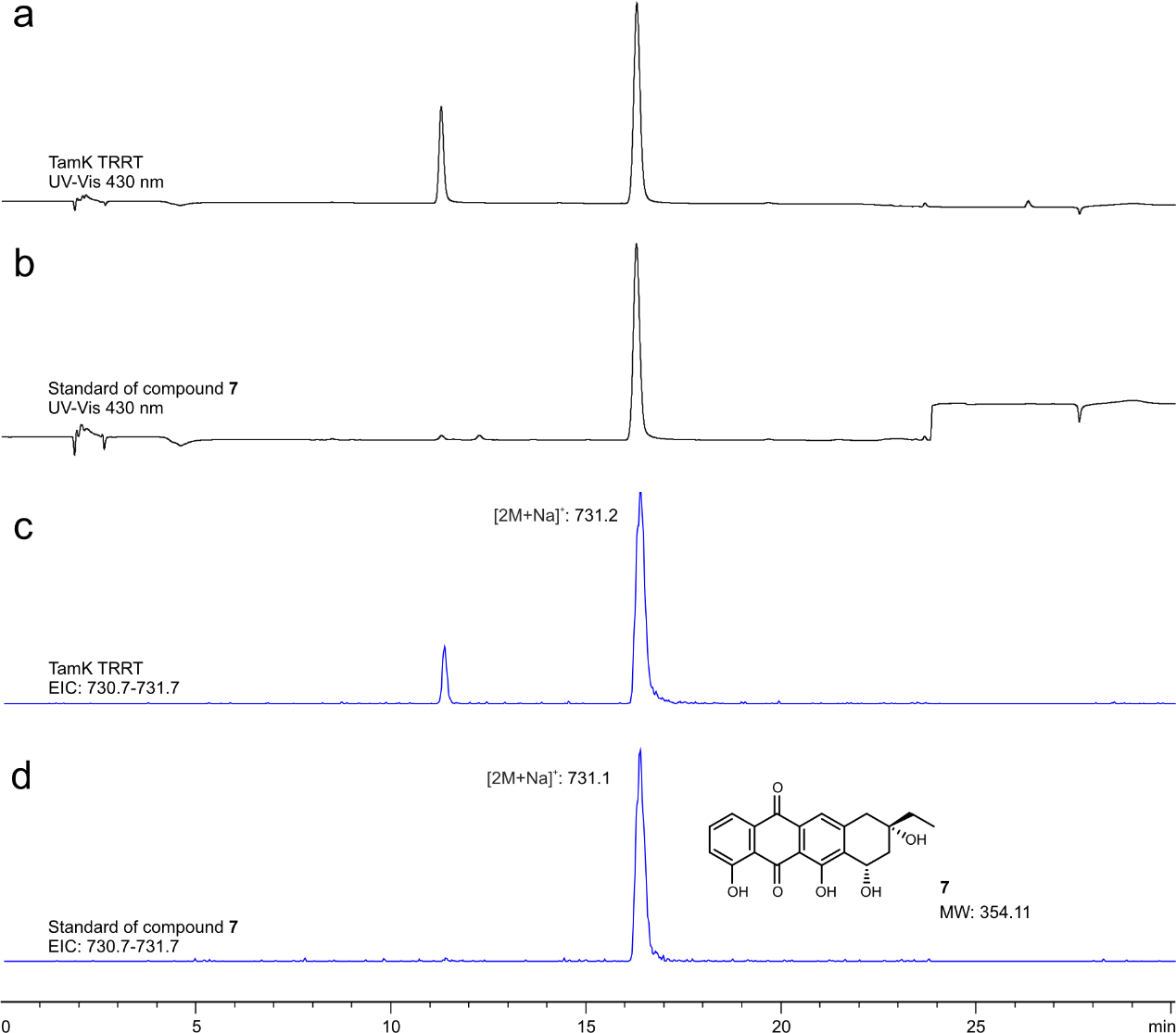


**Fig. S10. LC-MS analysis of the hydrolysed enzymatic reaction product 15 in comparison to authentic standard 7. a,** UV-Vis chromatogram trace recorded at 430 nm. Enzymatic reaction of chimeric TamK TRRT with **6** as a substrate was performed to obtain **15**, from which the glycosidic units were hydrolysed to obtain **7** (RT 16.3 min). The peak appearing at 11.3 min is a hydrolysis by-product. **b,** UV-Vis chromatogram trace recorded at 430 nm. The enzymatic reaction of native TamK with **4** as a substrate was performed to obtain the authentic standard **7**. **c,** Extracted ion chromatogram trace in positive mode of TamK TRRT products (**a**). **d,** Extracted ion chromatogram trace in positive mode of TamK product **(b)**. Products are observed as sodium adducts [2M+Na]^+^ under the conditions used, giving consistently values of 2M + 22.99.


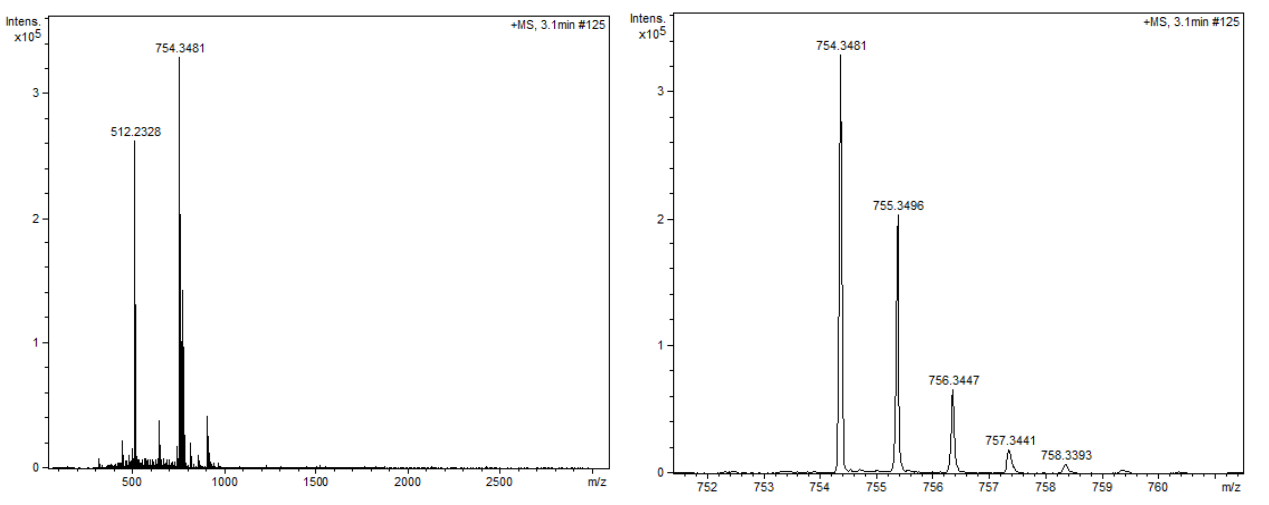


**Fig. S11. HR-MS spectrum of 15**. ESI m/z [M+H]^+^, ESI+ obs. 754.3481, calc. 754.3433.


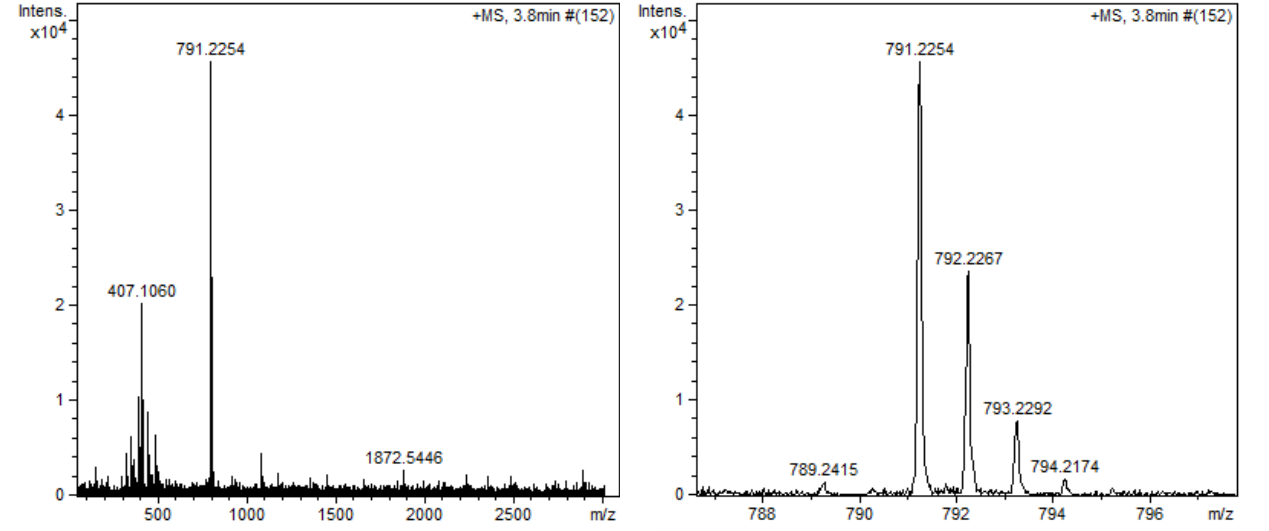


**Fig. S12. HR-MS spectrum of 13.** ESI m/z [2M+Na]^+^, ESI+ obs. 791.2254, calc. 791.2310


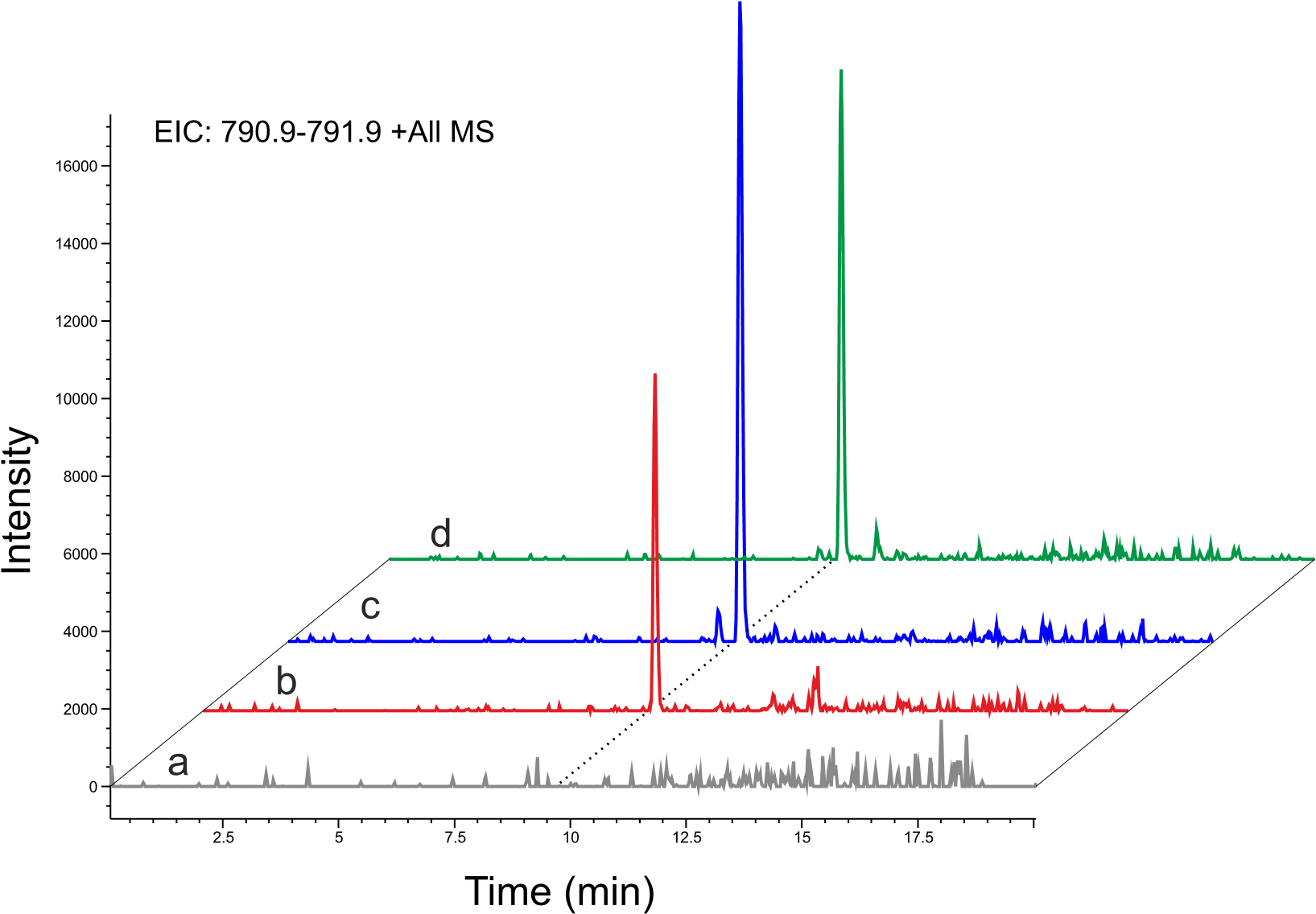


**Fig. S13. Analysis of DnrK RTTR reaction with 4 to yield 13.** **a**, Standard of compound **4.** **b,** Standard of compound **13**. DnrK and RdmB reactions were performed on **5** to obtain **14**, which was subsequently acid hydrolysed to yield the standard of compound **13.** **c**, DnrK RTTR reaction products with **4** as a substrate**.** **d,** Mixture of DnrK RTTR reaction products (**c**) and standard of compound **13** (**b**). All chromatograms are shown with the extracted ion chromatogram (EIC) for **13**. Products are observed as sodium adducts [2M+Na]^+^.


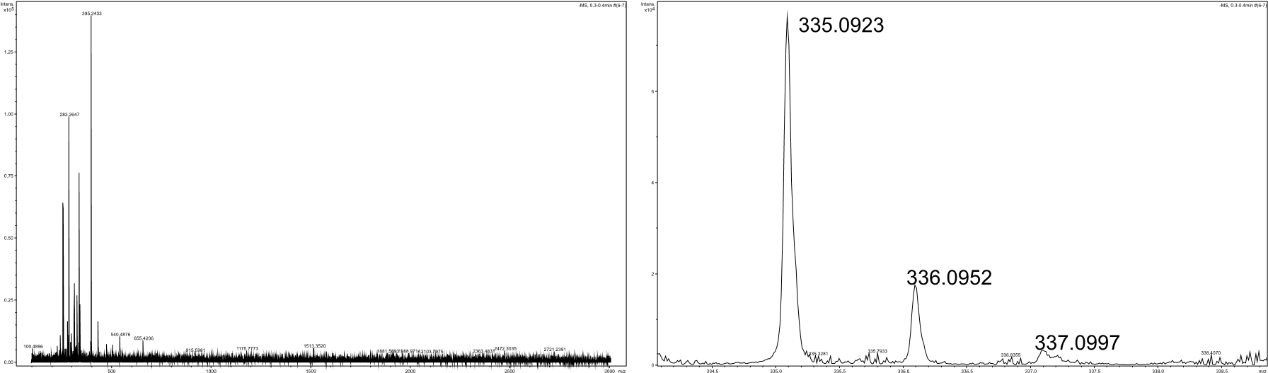


**Fig. S14. HR-MS spectrum of 16.** ESI m/z [M-H]-, ESI- obs. 335.0923, calc. 335.0925.

**Fig. S15. Main HMBC and COSY correlations for 16.** HMBC correlations are presented with blue arrows and COSY correlations with pink lines.


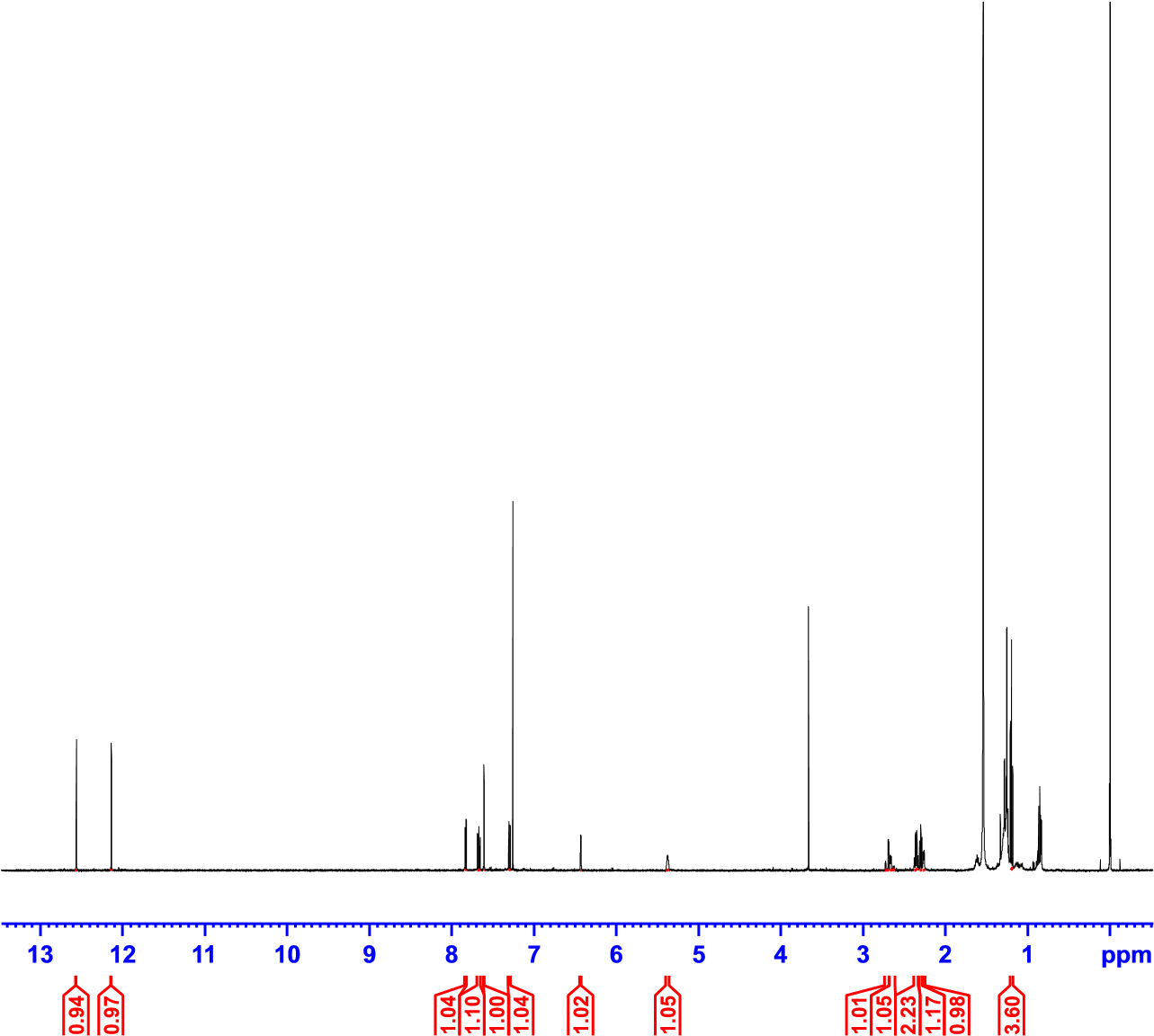


**Fig. S16. 1H spectrum of 16 in CDCl3.**


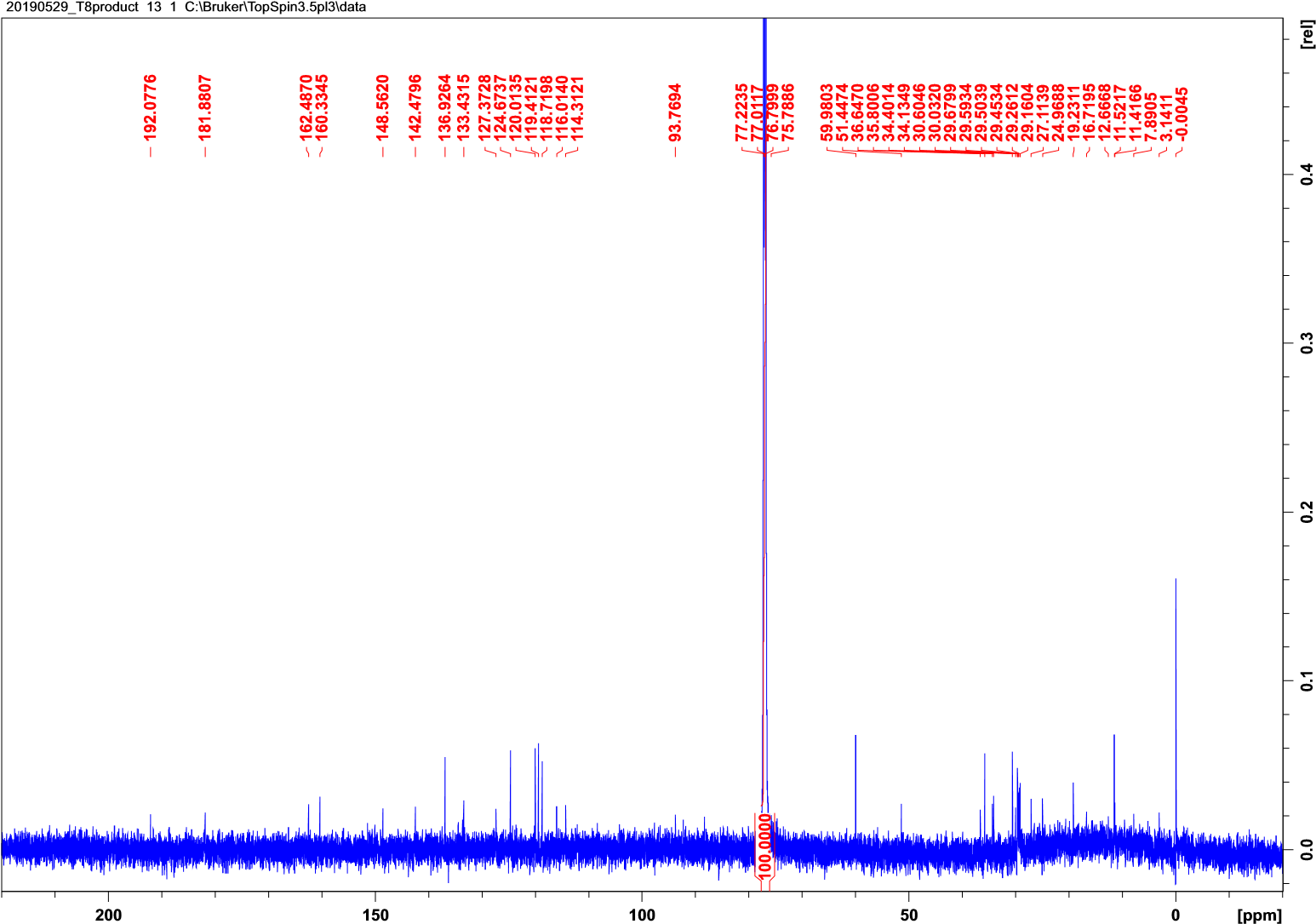


**Fig. S17. 13C spectrum of 16 in CDCl3.**


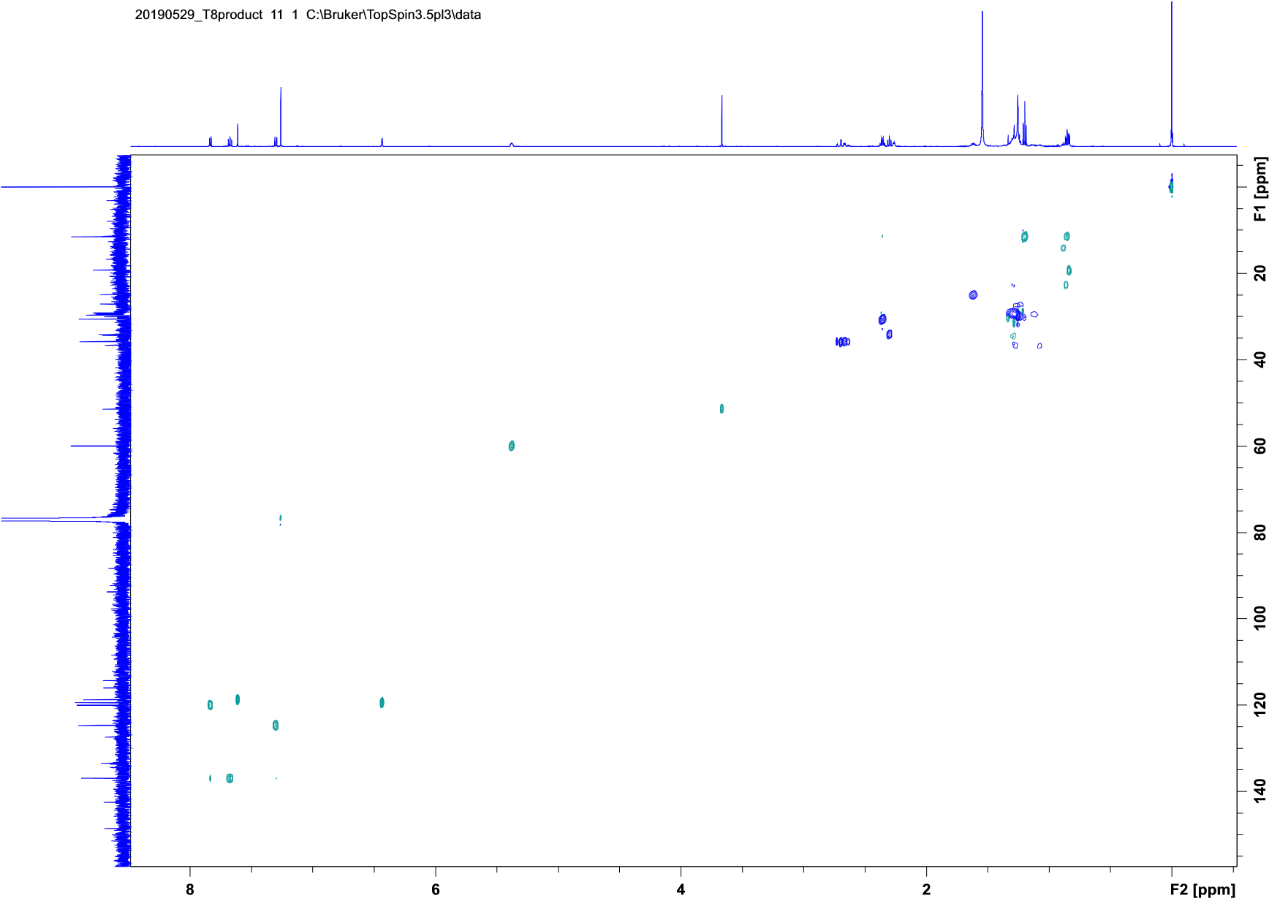


**Fig. S18. HSQCDE spectrum of 16 in CDCl3.**


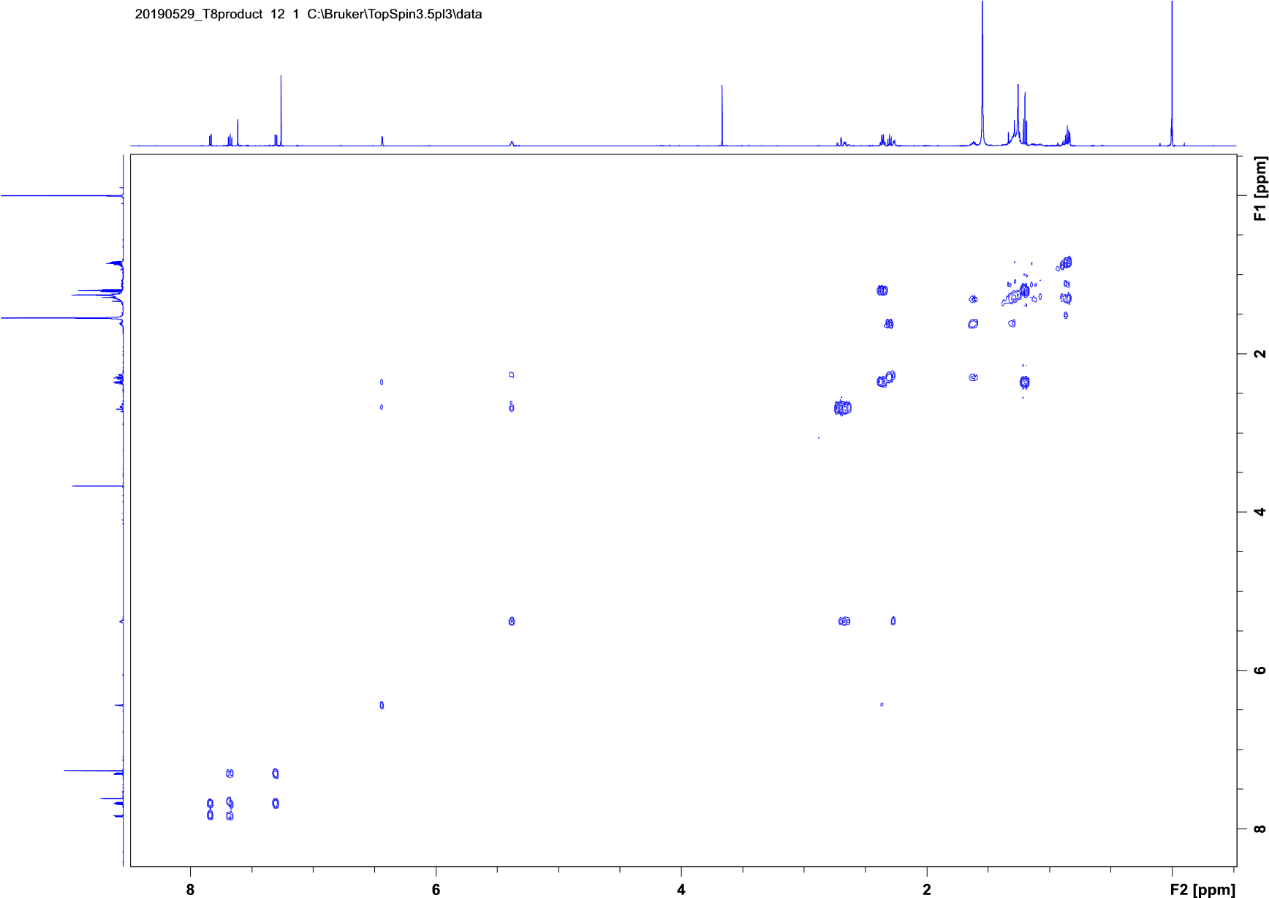


**Fig. S19. COSY spectrum of 16 in CDCl3.**


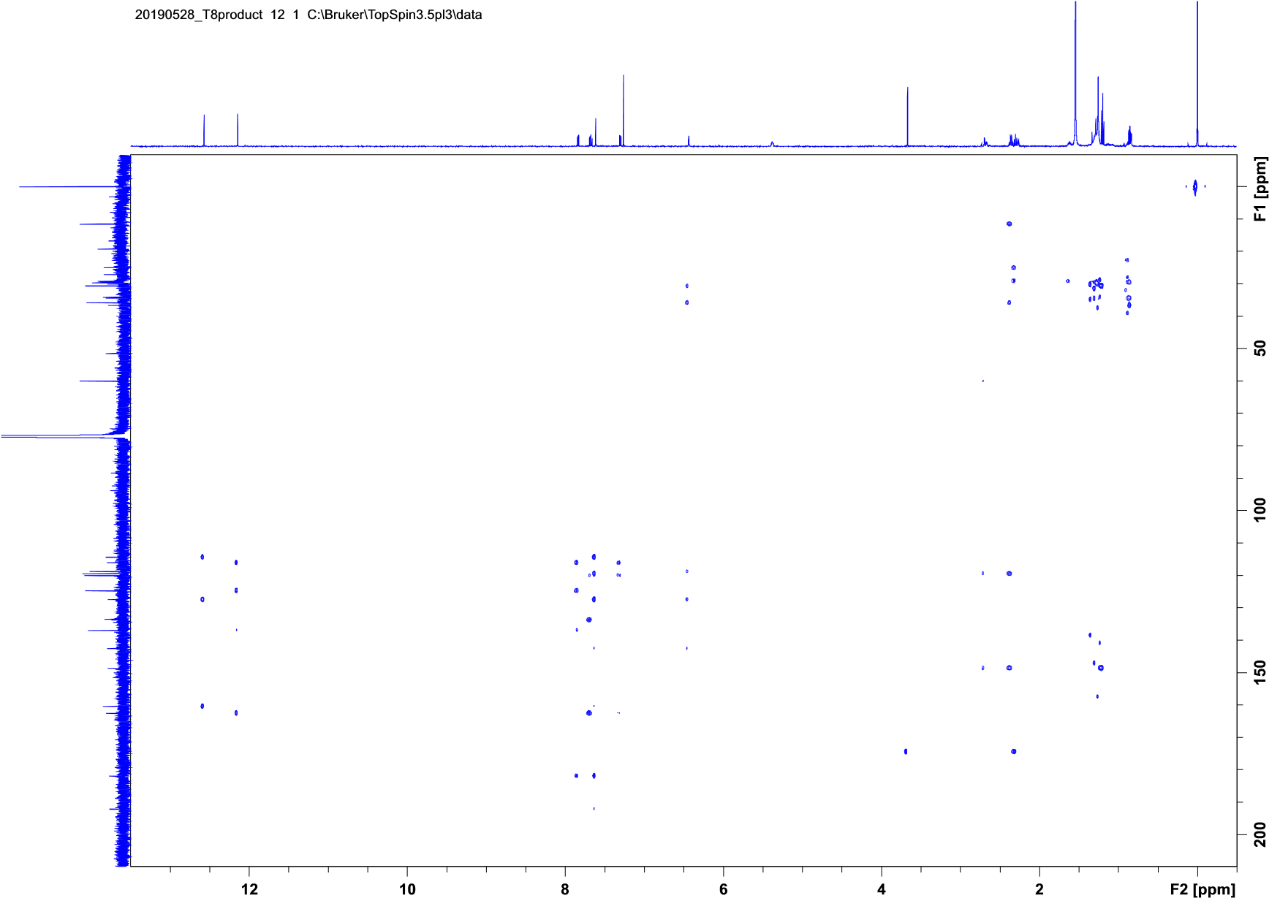


**Fig. S20. HMBC spectrum of 16 in CDCl3.**

**
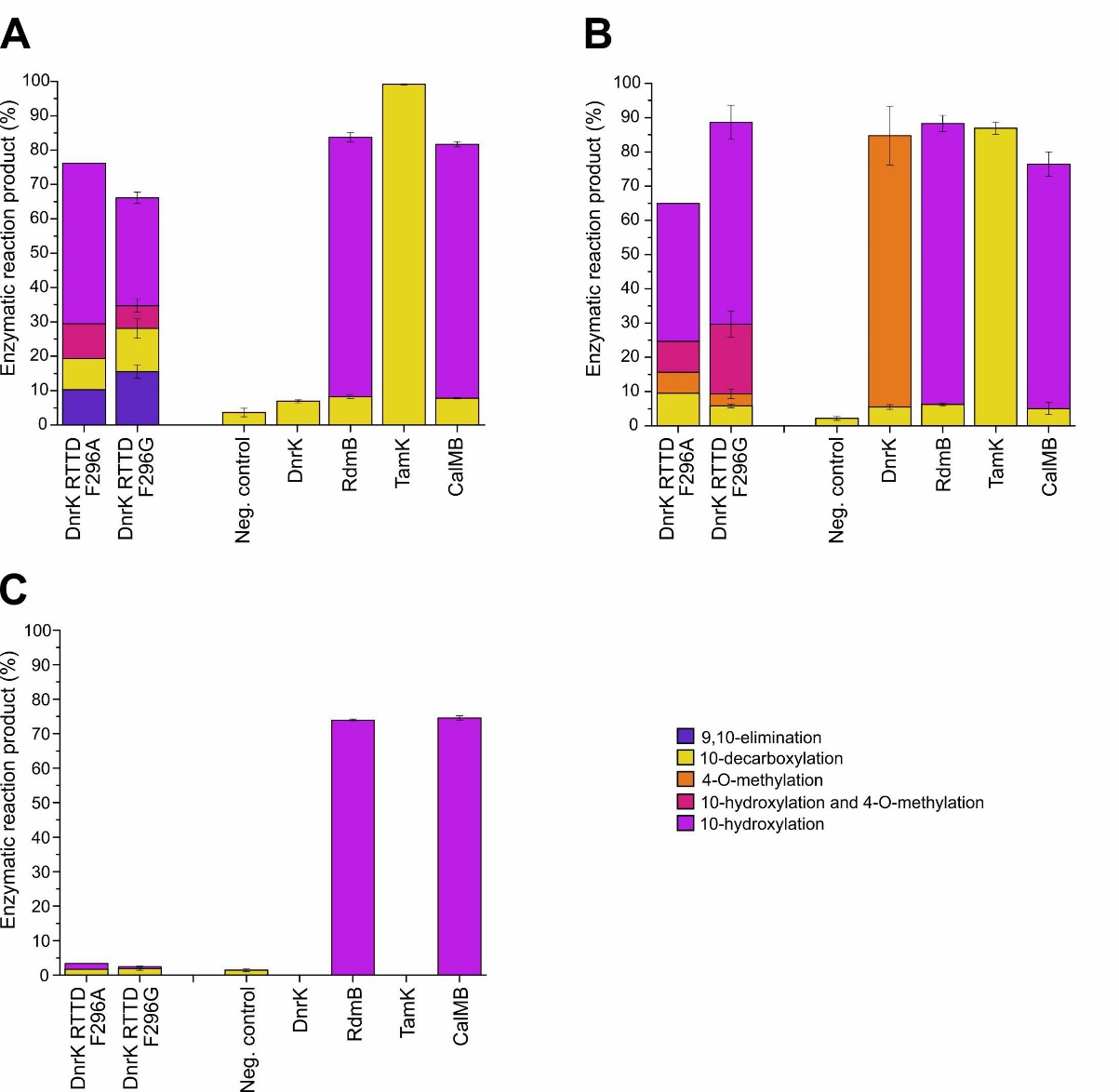
**

**Fig. S21. Comparison of enzymatic activities of DnrK RTTD F296A and DnrK RTTD F296G with anthracycline substrates.** Enzymatic reaction products with (A) **4**, (B) **5** and (C) **6** as substrates. The reaction product yields are shown as mean value ± standard deviation and were calculated from HPLC chromatogram traces by normalized peaks areas. The overall percentages may not add up to 100 % in all samples meaning that some unreacted substrate remained in the reaction mixture.


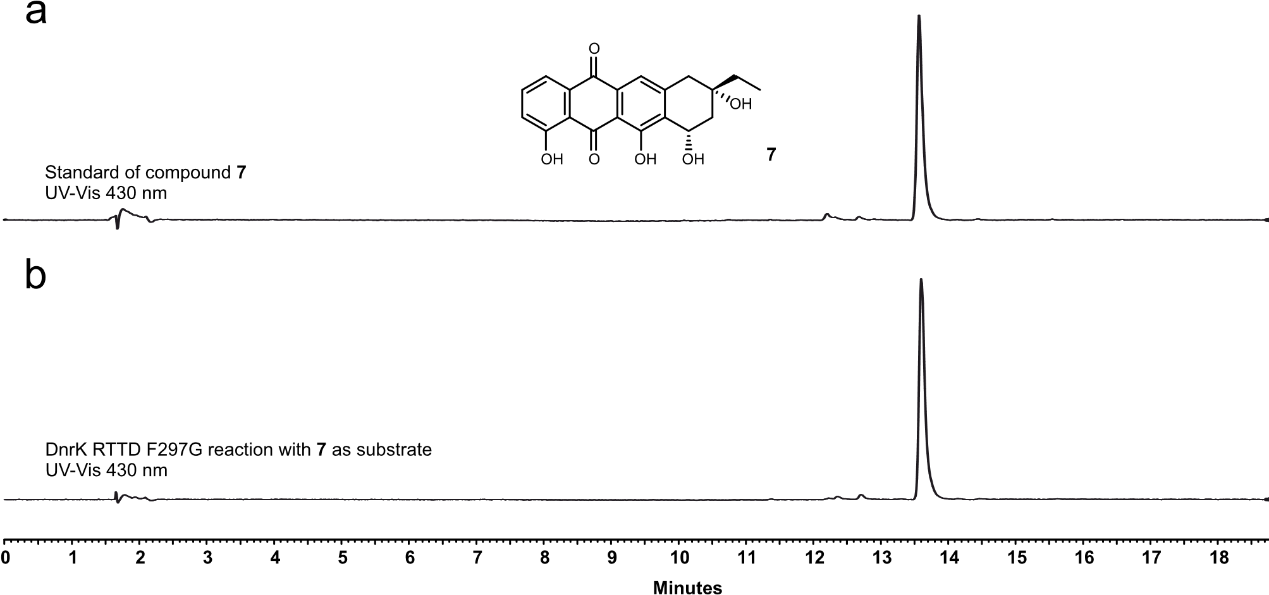


**Fig. S22. HPLC analysis of elimination reaction substrate. a,** UV-Vis chromatogram trace recorded at 430 nm for **7**. Standard of compound **7** was obtained through enzymatic reaction of TamK with **4** as a substrate. **b,** UV-Vis chromatogram trace recorded at 430 nm for DnrK RTTD F297G enzymatic reaction products with **7** as a substrate. No elimination reaction was observed.


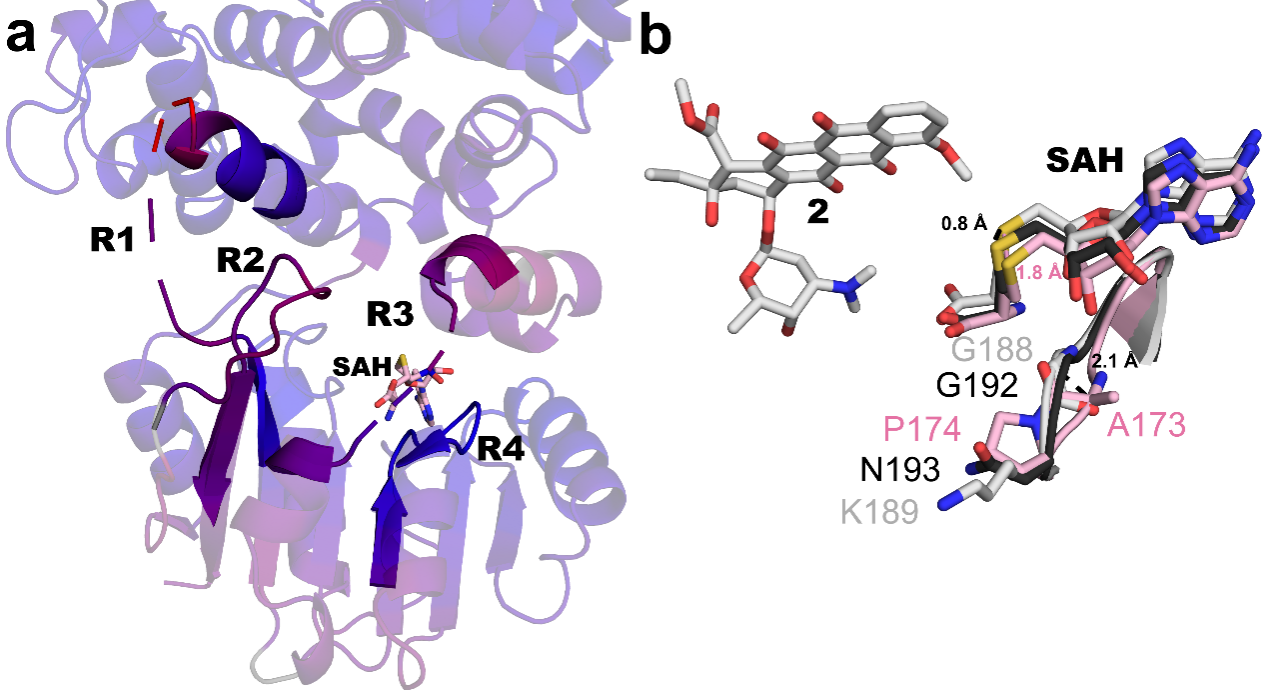


**Fig. S23. TamK structure and changes in region R4 and SAM binding. a,** The overall fold of TamK was aligned against DnrK colored by RMSD, with blue regions indicating a high degree of conservation and pink regions showing a higher variation between the two structures. The absence of substrate led to lack of density to build loops belonging to regions R1 and R3. **b,** Comparing the SAM binding region R4 of DnrK WT (white) (PDBID: 1TW2), RdmB (black) (PDBID: 1R00) and TamK WT (pink). The presence of P174 leads to a 2.1 Å rearrangement of the loop of region R4, leading to a repositioning of SAM (SAH in the figure). This movement is sufficient to prevent correct position of the cofactor in order to catalyse 4-O-methylation of the substrate.


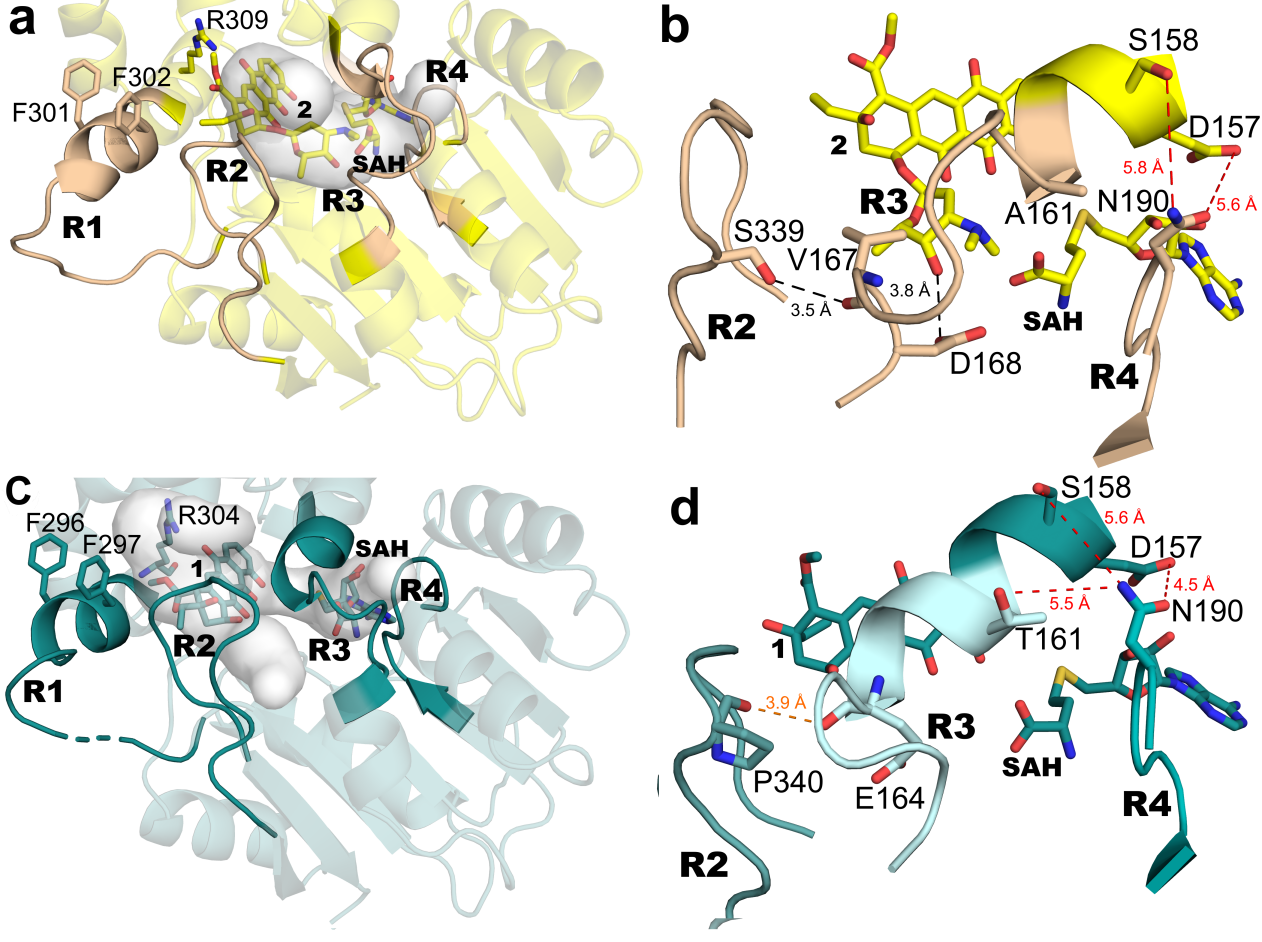


**Fig. S24.** **Comparison between DnrK RTTR and DnrK RTCR. a,** Overall fold of DnrK RTCR, crystallized in a closed conformation as evidenced by the CAVER analysis (light grey). The aromatic residues from region R1 prevent access to bulk solvent to the active site. R3 from CalMB shows the formation of a loop instead of the more common helix conformation. **b,** Closer observation of the inter-region interactions show a single hydrogen bond between S339 from R2 and the main chain of V167 in R3. D168 (R3) is seen interacting with the sugar moiety of the substrate. Similarly to DnrK RTTR (and DnrK WT) there is no hydrogen bonding possible between regions R3 and R4. **c,** Overall fold of DnrK RTTR. **d,** Inter-region interactions of DnrK RTTR.


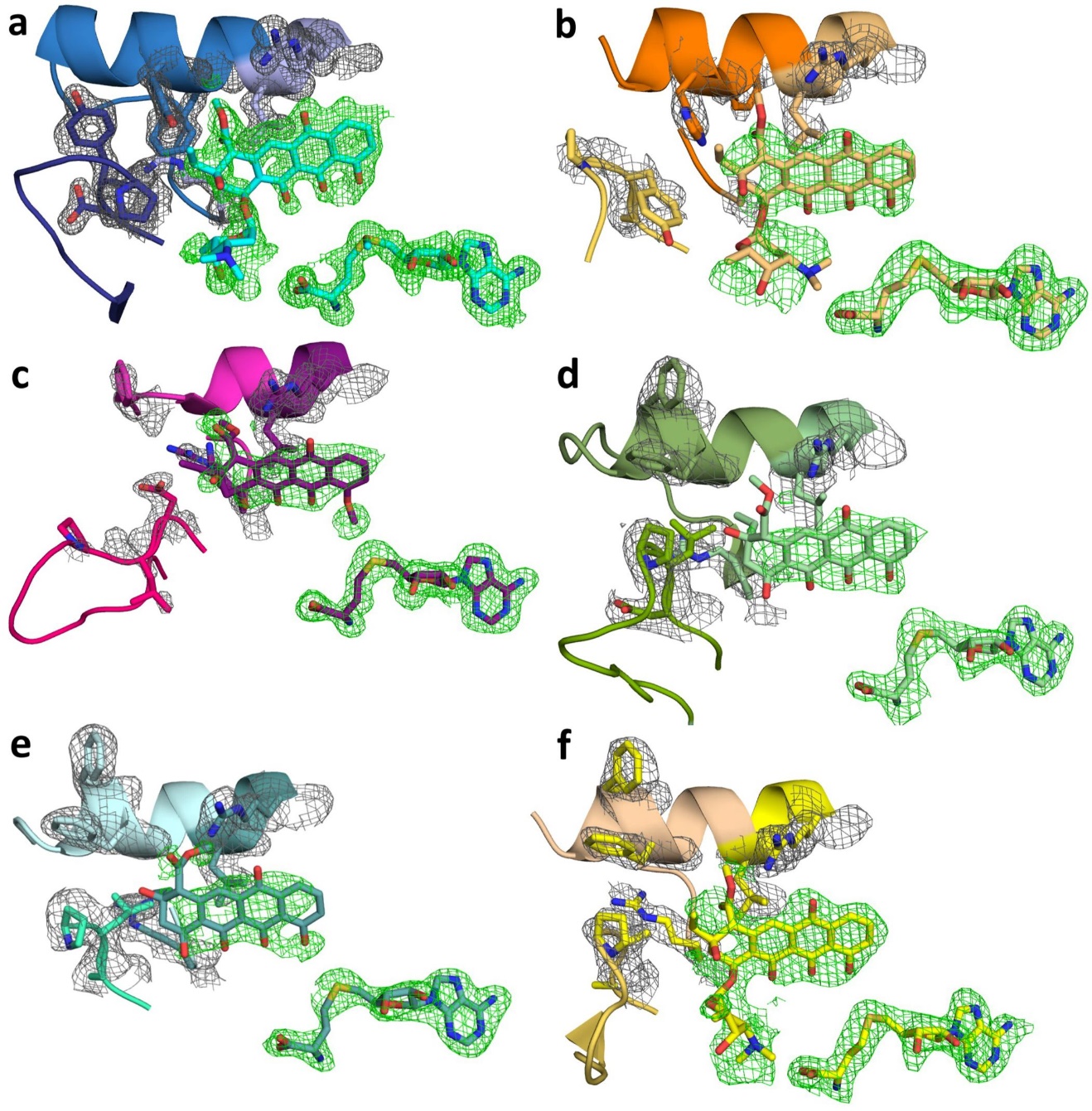


**Fig. S25. Fo-Fc electron density maps of chimeras with their substrate.** All Fo-Fc omit maps, shown in green, are contoured at 2.0 σ. 2Fo-Fc maps of relevant residues from regions R1 and R2, are contoured at 1.0 σ and shown in grey. **a,** DnrK TDDD (PDBID: 7PHD), crystallized with **2** and SAH. **b,** DnrK CDDD (PDBID: 7OWB), crystallized with **2** and SAH. **c**, DnrK RTTD F297G (PDBID: 7PGJ), crystallized with **1** and SAM. However, the 4-O-methylation reaction occurred in situ during crystallization and the reaction product is observed in the omit map. **d**, DnrK RTTD (PDBID: 7PGA), crystallized with **1** and SAH. **e**, DnrK RTTR (PDBID: 7PHF), crystallized with **1** and SAH. **f,** DnrK RTCR (PDBID: 7OY1), crystallized with **2** and SAH.

**Supplementary Tables**

**Table S1. Chimeric sequences being studied.** Amino acid sequences of the enzyme chimeras under study are presented, with regions under study in bold. The single point mutation introduced in the chimera DnrK RTTD F297G is indicated in red.

| **Protein name** | **Amino acid sequence** |
| --- | --- |
| DnrK WT | MAHHHHHHHRSTAEPTVAARPQQIDALRTLIRLGSLHTPMVV RTAATLRLVDHILAGARTVKALAARTDTRPEALLRLIRHLVA IGLLEEDAPGEFVPTEVGELLADDHPAAQRAWHDLTQAVARA DISFTRLPDAIRTGRPTYESIYGKPFYEDLAGRPDLRASFDS L**LACDQDVAFD**APAAAYDWTNVRHVL**DVGGGKG**GFAAAIARR APHVSATVLEMAGTVDTARSYLKDEGLSDRVDVVEGDFFEPL PRKADAIILSFVLLNWPDHDAVRILTRCAEALEPGGRILIHE R**DDLHENSFNEQFTE**LDLRMLVFLGGALRTREKWDGLAASAG LVVEEVR**QLPSPTIPYDLS**LLVLAPAATGA |
| DnrK RDDD | MAHHHHHHHRSTAEPTVAARPQQIDALRTLIRLGSLHTPMVV RTAATLRLVDHILAGARTVKALAARTDTRPEALLRLIRHLVA IGLLEEDAPGEFVPTEVGELLADDHPAAQRAWHDLTQAVARA DISFTRLPDAIRTGRPTYESIYGKPFYEDLAGRPDLRASFDS LLACDQDVAFDAPAAAYDWTNVRHVLDVGGGKGGFAAAIARR APHVSATVLEMAGTVDTARSYLKDEGLSDRVDVVEGDFFEPL PRKADAIILSFVLLNWPDHDAVRILTRCAEALEPGGRILIHE R**ADVEGDGADRFFSTL**LDLRMLVFLGGALRTREKWDGLAASA GLVVEEVRQLPSPTIPYDLSLLVLAPAATGA |
| DnrK CDDD | MAHHHHHHHRSTAEPTVAARPQQIDALRTLIRLGSLHTPMVV RTAATLRLVDHILAGARTVKALAARTDTRPEALLRLIRHLVA IGLLEEDAPGEFVPTEVGELLADDHPAAQRAWHDLTQAVARA DISFTRLPDAIRTGRPTYESIYGKPFYEDLAGRPDLRASFDS LLACDQDVAFDAPAAAYDWTNVRHVLDVGGGKGGFAAAIARR APHVSATVLEMAGTVDTARSYLKDEGLSDRVDVVEGDFFEPL PRKADAIILSFVLLNWPDHDAVRILTRCAEALEPGGRILIHE R**AEPSPDETTSTADLHFSL**LDLRMLVFLGGALRTREKWDGLA ASAGLVVEEVRQLPSPTIPYDLSLLVLAPAATGA |
| DnrK TDDD | MAHHHHHHHRSTAEPTVAARPQQIDALRTLIRLGSLHTPMVV RTAATLRLVDHILAGARTVKALAARTDTRPEALLRLIRHLVA IGLLEEDAPGEFVPTEVGELLADDHPAAQRAWHDLTQAVARA DISFTRLPDAIRTGRPTYESIYGKPFYEDLAGRPDLRASFDS LLACDQDVAFDAPAAAYDWTNVRHVLDVGGGKGGFAAAIARR APHVSATVLEMAGTVDTARSYLKDEGLSDRVDVVEGDFFEPL PRKADAIILSFVLLNWPDHDAVRILTRCAEALEPGGRILIHE R**AEAPSGGTRTSDLYFSV**LDLRMLVFLGGALRTREKWDGLAA SAGLVVEEVRQLPSPTIPYDLSLLVLAPAATGA |
| DnrK DDDT | MAHHHHHHHRSTAEPTVAARPQQIDALRTLIRLGSLHTPMVV RTAATLRLVDHILAGARTVKALAARTDTRPEALLRLIRHLVA IGLLEEDAPGEFVPTEVGELLADDHPAAQRAWHDLTQAVARA DISFTRLPDAIRTGRPTYESIYGKPFYEDLAGRPDLRASFDS LLACDQDVAFDAPAAAYDWTNVRHVL**DVGGAPG**GFAAAIARR APHVSATVLEMAGTVDTARSYLKDEGLSDRVDVVEGDFFEPL PRKADAIILSFVLLNWPDHDAVRILTRCAEALEPGGRILIHE RDDLHENSFNEQFTELDLRMLVFLGGALRTREKWDGLAASAG LVVEEVRQLPSPTIPYDLSLLVLAPAATGA |
| DnrK DDDR | MAHHHHHHHRSTAEPTVAARPQQIDALRTLIRLGSLHTPMVV RTAATLRLVDHILAGARTVKALAARTDTRPEALLRLIRHLVA IGLLEEDAPGEFVPTEVGELLADDHPAAQRAWHDLTQAVARA DISFTRLPDAIRTGRPTYESIYGKPFYEDLAGRPDLRASFDS LLACDQDVAFDAPAAAYDWTNVRHVL**DVGGGNG**GFAAAIARR APHVSATVLEMAGTVDTARSYLKDEGLSDRVDVVEGDFFEPL PRKADAIILSFVLLNWPDHDAVRILTRCAEALEPGGRILIHE RDDLHENSFNEQFTELDLRMLVFLGGALRTREKWDGLAASAG LVVEEVRQLPSPTIPYDLSLLVLAPAATGA |
| DnrK DDCD | MAHHHHHHHRSTAEPTVAARPQQIDALRTLIRLGSLHTPMVV RTAATLRLVDHILAGARTVKALAARTDTRPEALLRLIRHLVA IGLLEEDAPGEFVPTEVGELLADDHPAAQRAWHDLTQAVARA DISFTRLPDAIRTGRPTYESIYGKPFYEDLAGRPDLRASFDS L**MATEEEAVDE**APAAAYDWTNVRHVLDVGGGKGGFAAAIARR APHVSATVLEMAGTVDTARSYLKDEGLSDRVDVVEGDFFEPL PRKADAIILSFVLLNWPDHDAVRILTRCAEALEPGGRILIHE RDDLHENSFNEQFTELDLRMLVFLGGALRTREKWDGLAASAG LVVEEVRQLPSPTIPYDLSLLVLAPAATGA |
| DnrK DCCD | MAHHHHHHHRSTAEPTVAARPQQIDALRTLIRLGSLHTPMVV RTAATLRLVDHILAGARTVKALAARTDTRPEALLRLIRHLVA IGLLEEDAPGEFVPTEVGELLADDHPAAQRAWHDLTQAVARA DISFTRLPDAIRTGRPTYESIYGKPFYEDLAGRPDLRASFDS L**MATEEEAVDE**APAAAYDWTNVRHVLDVGGGKGGFAAAIARR APHVSATVLEMAGTVDTARSYLKDEGLSDRVDVVEGDFFEPL PRKADAIILSFVLLNWPDHDAVRILTRCAEALEPGGRILIHE RDDLHENSFNEQFTELDLRMLVFLGGALRTREKWDGLAASAG LVVEEVR**PITSPVVPFDFC**LLVLAPAATGA |
| DnrK DTTD | MAHHHHHHHRSTAEPTVAARPQQIDALRTLIRLGSLHTPMVV RTAATLRLVDHILAGARTVKALAARTDTRPEALLRLIRHLVA IGLLEEDAPGEFVPTEVGELLADDHPAAQRAWHDLTQAVARA DISFTRLPDAIRTGRPTYESIYGKPFYEDLAGRPDLRASFDS L**MTTREDTAFA**APAAAYDWTNVRHVLDVGGGKGGFAAAIARR APHVSATVLEMAGTVDTARSYLKDEGLSDRVDVVEGDFFEPL PRKADAIILSFVLLNWPDHDAVRILTRCAEALEPGGRILIHE RDDLHENSFNEQFTELDLRMLVFLGGALRTREKWDGLAASAG LVVEEVR**GPLVSPNVPLDSC**LLVLAPAATGA |
| DnrK RTTD | MAHHHHHHHRSTAEPTVAARPQQIDALRTLIRLGSLHTPMVV RTAATLRLVDHILAGARTVKALAARTDTRPEALLRLIRHLVA IGLLEEDAPGEFVPTEVGELLADDHPAAQRAWHDLTQAVARA DISFTRLPDAIRTGRPTYESIYGKPFYEDLAGRPDLRASFDS L**MTTREDTAFA**APAAAYDWTNVRHVLDVGGGKGGFAAAIARR APHVSATVLEMAGTVDTARSYLKDEGLSDRVDVVEGDFFEPL PRKADAIILSFVLLNWPDHDAVRILTRCAEALEPGGRILIHE R**ADVEGDGADRFFSTL**LDLRMLVFLGGALRTREKWDGLAASA GLVVEEVR**GPLVSPNVPLDSC**LLVLAPAATGA |
| DnrK RTTD F297G | MAHHHHHHHRSTAEPTVAARPQQIDALRTLIRLGSLHTPMVV RTAATLRLVDHILAGARTVKALAARTDTRPEALLRLIRHLVA IGLLEEDAPGEFVPTEVGELLADDHPAAQRAWHDLTQAVARA DISFTRLPDAIRTGRPTYESIYGKPFYEDLAGRPDLRASFDS L**MTTREDTAFA**APAAAYDWTNVRHVLDVGGGKGGFAAAIARR APHVSATVLEMAGTVDTARSYLKDEGLSDRVDVVEGDFFEPL PRKADAIILSFVLLNWPDHDAVRILTRCAEALEPGGRILIHE R**ADVEGDGADRFGSTL**LDLRMLVFLGGALRTREKWDGLAASA GLVVEEVR**GPLVSPNVPLDSC**LLVLAPAATGA |
| DnrK RTTR | MAHHHHHHHRSTAEPTVAARPQQIDALRTLIRLGSLHTPMVV RTAATLRLVDHILAGARTVKALAARTDTRPEALLRLIRHLVA IGLLEEDAPGEFVPTEVGELLADDHPAAQRAWHDLTQAVARA DISFTRLPDAIRTGRPTYESIYGKPFYEDLAGRPDLRASFDS L**MTTREDTAFA**APAAAYDWTNVRHVL**DVGGGNG**GFAAAIARR APHVSATVLEMAGTVDTARSYLKDEGLSDRVDVVEGDFFEPL PRKADAIILSFVLLNWPDHDAVRILTRCAEALEPGGRILIHE R**ADVEGDGADRFFSTL**LDLRMLVFLGGALRTREKWDGLAASA GLVVEEVR**GPLVSPNVPLDSC**LLVLAPAATGA |
| DnrK RTCR | MAHHHHHHHRSTAEPTVAARPQQIDALRTLIRLGSLHTPMVV RTAATLRLVDHILAGARTVKALAARTDTRPEALLRLIRHLVA IGLLEEDAPGEFVPTEVGELLADDHPAAQRAWHDLTQAVARA DISFTRLPDAIRTGRPTYESIYGKPFYEDLAGRPDLRASFDS L**MATEEEAVDE**APAAAYDWTNVRHVL**DVGGGNG**GFAAAIARR APHVSATVLEMAGTVDTARSYLKDEGLSDRVDVVEGDFFEPL PRKADAIILSFVLLNWPDHDAVRILTRCAEALEPGGRILIHE R**ADVEGDGADRFFSTL**LDLRMLVFLGGALRTREKWDGLAASA GLVVEEVR**GPLVSPNVPLDSC**LLVLAPAATGA |
| DnrK RCCR | MAHHHHHHHRSTAEPTVAARPQQIDALRTLIRLGSLHTPMVV RTAATLRLVDHILAGARTVKALAARTDTRPEALLRLIRHLVA IGLLEEDAPGEFVPTEVGELLADDHPAAQRAWHDLTQAVARA DISFTRLPDAIRTGRPTYESIYGKPFYEDLAGRPDLRASFDS L**MATEEEAVDE**APAAAYDWTNVRHVL**DVGGGNG**GFAAAIARR APHVSATVLEMAGTVDTARSYLKDEGLSDRVDVVEGDFFEPL PRKADAIILSFVLLNWPDHDAVRILTRCAEALEPGGRILIHE R**ADVEGDGADRFFSTL**LDLRMLVFLGGALRTREKWDGLAASA GLVVEEVR**PITSPVVPFDFC**LLVLAPAATGA |
| DnrK RTTT | MAHHHHHHHRSTAEPTVAARPQQIDALRTLIRLGSLHTPMVV RTAATLRLVDHILAGARTVKALAARTDTRPEALLRLIRHLVA IGLLEEDAPGEFVPTEVGELLADDHPAAQRAWHDLTQAVARA DISFTRLPDAIRTGRPTYESIYGKPFYEDLAGRPDLRASFDS L**MTTREDTAFA**APAAAYDWTNVRHVL**DVGGAPG**GFAAAIARR APHVSATVLEMAGTVDTARSYLKDEGLSDRVDVVEGDFFEPL PRKADAIILSFVLLNWPDHDAVRILTRCAEALEPGGRILIHE R**ADVEGDGADRFFSTL**LDLRMLVFLGGALRTREKWDGLAASA GLVVEEVR**GPLVSPNVPLDSC**LLVLAPAATGA |
|  |  |
| TamK WT | MAHHHHHHHRSSGTDAGTAGTAGTAGAGAGGDRQHVDALVRM SNLVTPMALRVAATLRLVDHLRAGATSADALADATGADADAL ARLMRHLAAAGVLEEPEPGHYAPTGLGDLLADDHPSRQRSWL DLDQAVGRADLTFLGLREAVRTGRPQYEARYGKPFWTDLSED DGLGASFDAL**MTTREDTAFA**APVAAYDWTRARHVL**DVGGAPG** GLLTAILRAAPEAHGTLLDLPGAAARTRERIAANGMDERIDV VGGDFFDELPVTADVVVLSFTLLNWSDPDALRILGRCRDALR PGGRIVLLER**AEAPSGGTRTSDLYFSV**LDMRMLVFLGGRVRT DREWADLAAAAGLDIVGKT**GPLVSPNVPLDSC**LWELAPR |
| TamK RRTT | MAHHHHHHHRSSGTDAGTAGTAGTAGAGAGGDRQHVDALVRM SNLVTPMALRVAATLRLVDHLRAGATSADALADATGADADAL ARLMRHLAAAGVLEEPEPGHYAPTGLGDLLADDHPSRQRSWL DLDQAVGRADLTFLGLREAVRTGRPQYEARYGKPFWTDLSED DGLGASFDALMTTREDTAFAAPVAAYDWTRARHVLDVGGAPG GLLTAILRAAPEAHGTLLDLPGAAARTRERIAANGMDERIDV VGGDFFDELPVTADVVVLSFTLLNWSDPDALRILGRCRDALR PGGRIVLLER**ADVEGDGADRFFSTL**LDMRMLVFLGGRVRTDR EWADLAAAAGLDIVGKTG**SGSTTLPFDFS**LWELAPR |
| TamK TRRT | MAHHHHHHHRSSGTDAGTAGTAGTAGAGAGGDRQHVDALVRM SNLVTPMALRVAATLRLVDHLRAGATSADALADATGADADAL ARLMRHLAAAGVLEEPEPGHYAPTGLGDLLADDHPSRQRSWL DLDQAVGRADLTFLGLREAVRTGRPQYEARYGKPFWTDLSED DGLGASFDAL**MSCDEDLAYE**APVAAYDWTRARHVLDVGGAPG GLLTAILRAAPEAHGTLLDLPGAAARTRERIAANGMDERIDV VGGDFFDELPVTADVVVLSFTLLNWSDPDALRILGRCRDALR PGGRIVLLERAEAPSGGTRTSDLYFSVLDMRMLVFLGGRVRT DREWADLAAAAGLDIVGKTG**SGSTTLPFDFS**LWELAPR |
| TamK RRRT | MAHHHHHHHRSSGTDAGTAGTAGTAGAGAGGDRQHVDALVRM SNLVTPMALRVAATLRLVDHLRAGATSADALADATGADADAL ARLMRHLAAAGVLEEPEPGHYAPTGLGDLLADDHPSRQRSWL DLDQAVGRADLTFLGLREAVRTGRPQYEARYGKPFWTDLSED DGLGASFDAL**MSCDEDLAYE**APVAAYDWTRARHVLDVGGAPG GLLTAILRAAPEAHGTLLDLPGAAARTRERIAANGMDERIDV VGGDFFDELPVTADVVVLSFTLLNWSDPDALRILGRCRDALR PGGRIVLLER**ADVEGDGADRFFSTL**LDMRMLVFLGGRVRTDR EWADLAAAAGLDIVGKTG**SGSTTLPFDFS**LWELAPR |

**Table S2. Compound 16 recorded in CDCl_3_.** ^1^H is recorded at 500 MHz and ^13^C at 151 MHz. The 2D measurements are done either in 500 MHz or 600 MHz for ^1^H and either at 126 MHz or 151 MHz for ^13^C. The signals are internally referenced to tetramethylsilane (TMS).

| Position | δ ppm | δ ppm, *J* Hz | HMBC | COSY |
| --- | --- | --- | --- | --- |
|  | 13C | ^1^H |  |  |
| 1 | 120.0 | 7.84 dd 1.0, 7.5 | C2, C3, C4a, C12 | H2 |
| 2 | 136.9 | 7.67 dd 7.5, 8.6 | C4, C12a | H1, H3 |
| 3 | 124.7 | 7.3 dd 1.0, 8.6 | C1, C4, C4a | H2 |
| 4 | 162.5 |  |  |  |
| 4-OH |  | 12.14 s | C3, C4, C4a |  |
| 4a | 116.0 |  |  |  |
| 5 | 192.0 |  |  |  |
| 5a | 114.3 |  |  |  |
| 6 | 160.4 |  |  |  |
| 6-OH |  | 12.57 s | C5a, C6, C6a, C10a |  |
| 6a | 127.4 |  |  |  |
| 7 | 60.0 | 5.38 ddd 2.5, 5.3, 5.9 |  | H7-OH, H8 |
| 7-OH |  | 2.27 d 5.3 |  | H7 |
| 8 | 35.8 | 2.64 ddd 1.3, 5.9, 18.6 | C9, C10 | H7, H8 |
| 8 |  | 2.71 dd 2.5, 18.6 |  | H7, H8 |
| 9 | 148.6 |  |  |  |
| 10 | 119.4 | 6.44 td 1.0, 1.3 | C6a, C8, C11, C13 | H8, H13 |
| 10a | 142.5 |  |  |  |
| 11 | 118.7 | 7.61 s | C5, C5a, C6a, C10, C12 |  |
| 11a | ND |  |  |  |
| 12 | 181.9 |  |  |  |
| 12a | 133.4 |  |  |  |
| 13 | 30.6 | 2.36 2H qd 1.0, 7.4 | C8, C9, C10, C14 | H9, H14 |
| 14 | 11.5 | 1.20 3H t 7.4 | C9, C13 | H13 |

s=singlet, d=douplet, t=triplet q=quartet, ND = no data, integrals are 1 unless otherwise stated

**Table S3. Data collection and refinement statistics**

|  | DnrK CDDD  (7OWB) | DnrK TDDD  (7PHD) | DnrK RTTD  (7PGA) | DnrK RTTD F297G  (7PGJ) | DnrK RTTR  (7PHF) | DrnK RTCR  (7OY1) | TamK  (7PG7) |
| --- | --- | --- | --- | --- | --- | --- | --- |
| **Data collection** |  |  |  |  |  |  |  |
| Space Goup | P 21 21 2 | P 1 21 1 | P 1 21 1 | C 2 2 21 | P 1 21 1 | P 1 21 1 | C 1 2 1 |
| Cell dimensions |  |  |  |  |  |  |  |
| *a, b, c* (Å) | 60.80, 103.14, 66.49 | 60.5, 101.3, 62.9 | 60.47, 102.64, 121.85 | 60.75, 124.95, 102.34 | 60.01, 102.42, 122.19 | 60.40, 105.69, 64.45 | 122.84, 39.69, 97.98 |
|   γ (°) | 90.00, 90.00, 90.00 | 90.00, 102.20, 90.00 | 90.03, 98.23, 90.00 | 90.00, 90.00, 90.00 | 90.00, 99.30, 90.00 | 90.00, 111.20, 90.00 | 90.00, 114.10, 90.00 |
| Resolution (Å) | 44.87 - 2.45 (2.51 - 2.45)) | 48.04 - 1.53 (1.59 - 1.53) | 45.89 - 2.77 (2.87 - 2.77) | 53.32 - 2.13 (2.21 - 2.13) | 47.13 - 2.21 (2.27 - 2.21) | 49.7- 2.39 (2.48 - 2.39 | 28.04 - 1.51 (1.57 - 1.51) |
| R_merge_ (%) | 13.5 (96.8) | 5.5 (58.8) | 11.6 (84.8) | 6.7 (18.4) | 11.2 (102.7) | 11.6 (24.2) | 22.62 (42.5) |
| *I/*σ*I* | 9.19 (1.56) | 11.18 (1.83) | 7.4 (1.1) | 22.40 (13.40) | 5.9 (0.9) | 8.3 (4.1) | 15.69 (2.24) |
| Completeness (%) | 99.12 (95.50) | 98.1 (98.0) | 98.4 (95.0) | 99.90 (100.00) | 99.3 (97.2) | 99.1 (96.0) | 96.51 (85.60) |
| Redundancy | 5.69 (4.83) | 2.69 (2.56) | 4.4 (4.3) | 6.6 (6.9) | 3.4 (3.4) | 3.4 (3.0) | 1.9 (1.8) |
| **Refinement** |  |  |  |  |  |  |  |
| No. reflections | 15808 (1483) | 109424 (10877) | 36928 (3701) | 22160 (2165) | 72307 (6991) | 29619 (2854) | 65666 (5771) |
| *R_work_*/*R_free_* | 0.25/0.26 | 0.22/0.24 | 0.20/0.25 | 0.17/ 0.22 | 0.23/0.27 | 0.23/0.27 | 0.20/0.22 |
| No. atoms |  |  |  |  |  |  |  |
| Protein | 2521 | 4772 | 10280 | 2553 | 9504 | 5113 | 2713 |
| Ligand/ion | 41 | 174 | 120 | 31 | 90 | 82 | 26 |
| Water | 32 | 334 | 141 | 261 | 223 | 264 | 245 |
| B-factors |  |  |  |  |  |  |  |
| Protein | 48.02 | 20.24 | 25.10 | 18.84 | 49.08 | 16.79 | 29.76 |
| Ligand/Ion | 34.98 | 31.61 | 29.31 | 27.06 | 40.49 | 37.39 | 25.20 |
| Water | 43.32 | 30.20 | 17.32 | 27.70 | 49.20 | 20.12 | 40.42 |
| R.m.s. deviations |  |  |  |  |  |  |  |
| Bond lengths (Å) | 0.011 | 0.010 | 0.005 | 0.008 | 0.008 | 0.009 | 0.007 |
| Bond angles (°) | 1.41 | 1.23 | 0.86 | 0.92 | 1.04 | 1.18 | 0.99 |

**Supplementary Information References**

1. F. Sievers, et al., Fast, scalable generation of high-quality protein multiple sequence alignments using Clustal Omega. Mol. Syst. Biol. 7, 539 (2011). [↑](#endnote-ref-1)
2. P. Gouet, E. Courcelle, ENDscript: A workflow to display sequence and structure information. Bioinformatics 18, 767–768 (2002). [↑](#endnote-ref-2)
